# Supplementary material for: Effectiveness of three delivery models for promoting access to pre-exposure prophylaxis in HIV-1 serodiscordant couples in Nigeria
Source: PLoS One. 2022 May 5;17(5):e0268011. doi: 10.1371/journal.pone.0268011 (PMC9070899; doi:10.1371/journal.pone.0268011)
Supplement: S1 File — (PDF) [file pone.0268011.s002.pdf]

03 May 2016

To Whom It May Concern:

RE: A Demonstration Project of Antiretroviral-based HIV-1 Prevention Among High-risk HIV-1 Serodiscordant Couples in Nigeria.

As project manager for the Pan African Clinical Trial Registry (www.pactr.org) database, it is my pleasure to inform you that your application to our registry has been accepted. Your unique identification number for the registry is **PACTR201605001602542**

Please be advised that your trial is registered under an initiative within our system that allow us to capture data of trials that are already in progress or completed. As such, your trial registration may not adhere to the mandates set forth by the International Committee of Medical Journal Editors for registration requirements, and it is your duty to be transparent to any journal that may ask about the retrospective status of your registration.

Please note you are responsible for updating your trial, or for informing us of changes to your trial. Additionally, please provide us with copies of your ethical clearance letters as we must have these on file (via email, post or fax) at your earliest convenience if you have not already done so.

Please do not hesitate to contact us at +27 21 938 0835 or email epienaar@mrc.ac.za should you have any questions.

Yours faithfully,

Elizabeth D Pienaar
www.pactr.org Project Manager
+27 021 938 0835

THE SOUTH AFRICAN MEDICAL RESEARCH COUNCIL
Cochrane South Africa | PO Box 19070, Tygerberg, 7505
Tel: +27 (0)21 938 0438 | Email: cochrane@mrc.co.za | Web: www.southafrica.cochrane.org
