# Supplementary material for: Effectiveness of three delivery models for promoting access to pre-exposure prophylaxis in HIV-1 serodiscordant couples in Nigeria
Source: PLoS One. 2022 May 5;17(5):e0268011. doi: 10.1371/journal.pone.0268011 (PMC9070899; doi:10.1371/journal.pone.0268011)
Supplement: S2 File — (PDF) [file pone.0268011.s003.pdf]

A Demonstration Project of Antiretroviral-based HIV-1 Prevention Among High-risk HIV-1 Serodiscordant Couples in Nigeria

In collaboration with

Georgetown University, London School of Health and Tropical Medicine, WHO, UNAIDS, Gilead

Sponsored by

Bill and Melinda Gates Foundation

Principal Investigator

John Idoko, MD

Co-Principal Investigator

Morenike O Ukpong, FWACS, MBA

Approved Operational Copy

An adaptation of The Partners PrEP Study Demonstration Project of Kenya

Approved Operational Copy

Table of Contents

Protocol Team Roster 5

Summary 7

1.0 Background And Rationale 9
- 1.1 ART and HIV-I transmission
- 1.2 Concern about PrEP and adherence
- 1.3 Use of ART for HIV-1 prevention in serodiscordant couples
- 1.4 Targeting antiretroviral-based prevention to highest-risk couples
- 1.5 Staged PrEP until ART initiation for HIV-1 prevention in couples
- 1.6 The need for demonstration projects
- 1.7 PrEP as part of a complete prevention package
- 1.8 The Quality Improvement (QI) approach to service delivery

2.0 Study Method 27

3.0 Objectives 28
- 3.1 Primary objectives
- 3.2 Secondary objectives

4.0 Population 32

5.0 Study Sites 33

6.0 Study Services 33

7.0 Eligibility 34
- 7.1 For heterosexual couples
- 7.2 For HIV-1 uninfected members of the couple (partner participants)

8.0 Sample Size 34

9.0 PrEP Medication 35

10.0 Recruitment 35

11.0 Study Procedures 36
- 11.1 Community education and mobilization
- 11.2 Study site preparation
- 11.3 Supply, storage and dispensing of PrEP drugs
- 11.4 Recruitment, screening and enrollment
- 11.5 Monitoring adherence and safety
- 11.6 Co-enrollment guidelines
- 11.7 Participant retention
- 11.8 Participant withdrawal
- 11.9 Adherence counseling and case management
- 11.10 Support for adherence
- 11.11 Support for HIV positive partners
- 11.12 Suspending or discontinuing PrEP
- 11.13 Concomitant medications

12.0 Safety 43

Approved Operational Copy

12.1 Adverse events and reporting requirements
12.2 Adverse event reporting
12.3 Serious adverse event (SAE) reporting
12.4 Safety monitoring

13.0 Data management and analysis 44
13.1 Data analysis
13.2 Data management

14.0 Ethical Considerations 45
14.1 Regulatory and ethical review
14.2 Informed consent
14.3 Risks
14.4 Benefits
14.5 Access to HIV-related care
14.6 Community involvement and consultation
14.7 Confidentiality
14.8 Study Discontinuation

15.0. Laboratory Considerations..48
15.1 Laboratory specimens
15.2 On site testing
15.3 Laboratory quality control and quality assurance procedures

16.0. Administrative Procedures 49
16.1 Protocol compliance
16.2 Protocol deviations and violations
16.3 Quality assurance
16.4 Study monitoring
16.5 Study records
16.6 Use of information and publications

Figures

Figure 1: Cost Effectiveness of long-term PrEP vs. early ART 16

Figure 2: Holt & Laury game ....
22

Tables

Table 1: Methods to elicit risk preferences
....23

Approved Operational Copy

Protocol Team Roster

National Agency for the Control of AIDS

Prof John Idoko
Principal Investigator
National Agency for the Control of AIDS
Ralph Sodeinde Street, Abuja

Morenike Oluwatoyin Ukpong
Co-Principal Investigator
Institute of Public Health
Obafemi Awolowo University
Ile-Ife

Dr Emmanuel Alhassan
Resource Mobilisation
National Agency for the Control of AIDS
Ralph Sodeinde Street, Abuja

Dr James Anenih
Research Coordination
National Agency for the Control of AIDS
Ralph Sodeinde Street, Abuja

Prof Ekanem
Protocol Statistician
University of Lagos
Akoka, Lagos

Dr Joseph Asonye Nnorom
Chembio Diagnostics Nigeria Limited,
House 9, A Close, 24 Crescent,
Gwarinpa Estate, Abuja.

Dr Rui Miguel Vaz
The WHO Representative
World Health Organization
Abuja

Dr Chidi Nweneka
Protocol Specialists
National Agency for the Control of AIDS
Ralph Sodeinde Street, Abuja

Dr Matthias Alaga
Statistics and Data Manager
National Agency for the Control of AIDS
Ralph Sodeinde Street, Abuja

Dr Kenneth Kalu
Grant Manager
National Agency for the Control of AIDS
Ralph Sodeinde Street, Abuja

Dr Kayode Ogungbemi
Monitoring and Evaluation
National Agency for the Control of AIDS
Ralph Sodeinde Street, Abuja

Community engagement

Dr Ali Onoja
African Health Project,
211 Neigbourhood Centre
Off Wole Soyinka Avenue,
Gwarinpa, Garki Abuja

Ms Florita Durueke
New HIV Vaccine and Microbicide Advocacy Society,
51/52 Ijaye Road, Ogba, Lagos

Mr Ibrahim Umoru
Community Services Manager
Hygeia Foundation
Elephant House
214, Broad Street, Lagos

Social Scientists

Mrs Nancin Dandem
APIN, Nigeria

Mrs Grace Kolawole,
APIN, JUTH, Nigeria.

Site Principal Investigators

Dr Kwasi Torpey
FHI360/SIDHAS
Abuja

Approved Operational Copy

Prof AS Sagay
Department of Obstetrics and Gynaecology
University of Jos

Dr Hadiza Kamofu
FHI360/SIDHAS project
Abuja

Ethics oversight
Mr Aminu Yakubu
National Health Research Ethics Committee
Federal Ministry of Health, Abuja

Regulatory oversight
Dr Beno Yakubu
Director, Clinical Evaluations and Trials Unit
National Agency for Food and Drug Administration and Control (NAFDAC)
Oshodi, Lagos

Study Monitors

Dr Oliver Ezechi
Project Monitor
Nigeria Institute of Medical Research
Lagos, Nigeria

Dr Kelly O'Reily
Independent Study Monitor
World Health Organisation,
Geneva

Collaborating Institutions

Dr Mark Dybul
Georgetown University
USA

Fern Terris-Prestholt
LSHTM, London

Aurelia Lepine
LSHTM, London

Representative
World Health Organization

Representative
UNAIDS

Representative
Bill and Melinda Gates Foundation

Representative
Gilead Sciences
333 Lakeside Drive
Foster City, CA 94404

Approved Operational Copy

Summary

Multiple studies have established that anti-retroviral drugs could be used to reduce the rate of infection in a population in two different ways, known by the acronyms TasP (Treatment as Prevention) and PrEP (Pre-Exposure Prophylaxis).

TasP targets people who are already infected with HIV-1, reducing their infectiousness in the population. Multiple studies have established that, after about the first six months of treatment, Anti-Retroviral Therapy (ART) reduces HIV-1 concentrations to undetectable levels in blood plasma and genital compartments. Additional studies have demonstrated that HIV transmission by an infected person is significantly reduced when that person adheres to ART well enough to maintain viral suppression.

PrEP targets people who are not infected with HIV-1. The PrEP strategy supplies a drug that, taken regularly by an uninfected person, reduces the probability of becoming infected on exposure to the HIV-1 virus. (A familiar similar strategy is the "sunday-sunday pills" used to prevent malaria.) Multiple studies have demonstrated that adherence to a PrEP regime works.

Although it will collect data relevant to efficacy, it is not the purpose of this study to establish the efficacy of TasP and PrEP. That work has been done and, for this study, the efficacy of these strategies is presumed established. The open issue with TasP and PrEP is how to implement the strategies in a large population as part of a comprehensive public health program aimed at preventing HIV infection. What are the barriers to uptake; that is, why would people hesitate to adopt a prevention strategy? What are the barriers to adherence; that is, what would prevent a person who has started PrEP or ART from continuing it? What are the most cost-effective ways of delivering, not just the drugs, but the counseling and support needed to make the strategies work? Should there be a separate program? Is it better done within an established program?

If this demonstration project is successful, we will have in hand a practical plan for the establishment of a comprehensive national program to prevent HIV infection in Nigeria.

The target population for the demonstration project will be heterosexual serodiscordant couples. Several studies have identified this as a priority population for this kind of intervention, but more than that, enough work has been done with this population in enough places that we now have sociological tools specific to them that we can use with some confidence. WHO, for example, is about to release guidelines for counseling serodiscordant couples about prevention; they will include ART and PrEP as prevention strategies. The study "Partner's PrEP," for another example, developed a simple and effective risk assessment tool that proved to be a good predictor of behaviors in this population. We intend to use it in this project to place participants.

The treatment strategy that will be used in this project adopt both TasP and PrEP. For a serodiscordant couple, PrEP and TasP work together for long-term prevention of HIV infection. ART, necessary for TasP, is not indicated for the infected partner until quite some time after initial diagnosis. Throughout this period, the uninfected partner is at elevated risk. However, if PrEP is adopted by the uninfected partner when the infected partner is diagnosed, that risk is significantly reduced. Eventually the infected partner begins ART

Approved Operational Copy

and sometime later achieves viral suppression. At this point as TasP strategy can be adopted and PrEP ended.

To start, a sample with a size of 600 heterosexual HIV-1 serodiscordant couples will be recruited for the study. PrEP would be administered through three sites selected from three States in Nigeria – Anambra, Cross River and Jos.

The project will have three primary goals:

- Construct a model that effectively delivers PrEP to serodiscordant couples. Within the context of this goal, the measure of effectiveness will be the number of new infections averted.
- Measure the cost effectiveness of the model. The measure of cost effectiveness will be cost per life-year gained and cost per infection averted
- Estimate what would be needed to scale up the delivery model to the national level.

Given the data and experience gained from the project, it should be possible to deliver a practical plan, complete with a budget estimate, to scale up PrEP and TasP, and make them part of a comprehensive national HIV prevention package.

Approved Operational Copy

1.0 Background and Rationale

After 30 years of the HIV-1 epidemic, novel and effective HIV-1 prevention strategies remain urgently needed, particularly those that are deliverable to and useable by high-risk populations. To achieve impact at the population level with strategies that are proven efficacious in controlled trials, effective delivery systems and high uptake are critical.[1] To lower the cost per averted infection, targeted delivery to those at highest risk for HIV-1 will be essential.

During the past 10 years, a growing scientific and advocacy interest in antiretroviral-based strategies for prevention of sexual HIV-1 transmission has developed, and antiretroviral-based HIV-1 prevention interventions are now among the most promising strategies for dramatically reducing the spread of HIV-1.[2] Antiretrovirals have the potential to be used for HIV-1 prevention as 1) antiretroviral treatment (ART) to reduce the infectiousness of HIV-1 infected persons (TasP) and 2) oral or topical pre-exposure prophylaxis (PrEP) for uninfected persons with repeated and ongoing HIV-1 exposure.[3]

1.1 ART and HIV-I transmission

ART reduces HIV-1 transmission by reducing HIV-1 plasma concentrations to undetectable levels within 6 months of initiation in the majority of persons[4] and seminal and cervicovaginal HIV-1 concentrations are also reduced to undetectable levels in most persons on ART.[5] Also, the use of peripartum ART is responsible for the remarkable success in virtually eliminating mother-to-child HIV-1 transmission in resource-rich settings.[6] The efficacy of antiretrovirals for the prevention of mother-to-child transmission of HIV-1, first demonstrated with peripartum zidovudine[7] showed the feasibility of preventing sexual acquisition of HIV-1 with PrEP. More recent studies have shown that

1 Merson M, Padian N, Coates TJ, Gupta GR, Bertozzi SM, Piot P, et al. Combination HIV prevention. Lancet 2008,372:1805-1806.

2Cohen MS, Gay C, Kashuba AD, Blower S, Paxton L. Narrative review: antiretroviral therapy to prevent the sexual transmission of HIV-1. Ann Intern Med 2007,146:591-601

3 Karim SS, Karim QA. Antiretroviral prophylaxis: a defining moment in HIV control. Lancet 2011,17:17; Padian NS, McCoy SI, Karim SS, Hasen N, Kim J, Bartos M, et al. HIV prevention transformed: the new prevention research agenda. Lancet 2011,378:269-278.

4 Phillips AN, Staszewski S, Weber R, Kirk O, Francioli P, Miller V, et al. HIV viral load response to antiretroviral therapy according to the baseline CD4 cell count and viral load. J Am Med Assoc 2001,286:2560-2567; Graham SM, Holte SE, Peshu NM, Richardson BA, Panteleeff DD, Jaoko WG, et al. Initiation of antiretroviral therapy leads to a rapid decline in cervical and vaginal HIV-1 shedding. AIDS 2007,21:501-507.

5 Graham SM, Holte SE, Peshu NM, Richardson BA, Panteleeff DD, Jaoko WG, et al. Initiation of antiretroviral therapy leads to a rapid decline in cervical and vaginal HIV-1 shedding. AIDS 2007,21:501-507; Gupta P, Mellors J, Kingsley L, Riddler S, Singh MK, Schreiber S, et al. High viral load in semen of human immunodeficiency virus type 1-infected men at all stages of disease and its reduction by therapy with protease and nonnucleoside reverse transcriptase inhibitors. J Virol 1997,71:6271-6275; Marcelin AG, Tubiana R, Lambert-Niclot S, Lefebvre G, Dominguez S, Bonmarchand M, et al. Detection of HIV-1 RNA in seminal plasma samples from treated patients with undetectable HIV-1 RNA in blood plasma. AIDS 2008,22:1677-1679; Cu-Uvin S, Caliendo AM. Genital tract HIV-1 RNA shedding among women with below detectable plasma viral load. AIDS 2011,25:880-881; Vernazza PL. Genital shedding of HIV-1 despite successful antiretroviral therapy. Lancet 2001,358:1564.

6 Mofenson LM. Can perinatal HIV infection be eliminated in the United States? J Am Med Assoc 1999,282:577-579.

7 Connor EM, Sperling RS, Gelber R, Kiselev P, Scott G, O'Sullivan MJ, et al. Reduction of maternal-infant transmission of human immunodeficiency virus type 1 with zidovudine treatment. Pediatric AIDS Clinical Trials Group Protocol 076 Study Group. N Engl J Med 1994,331:1173-1180.

Approved Operational Copy

post-natal antiretrovirals, provided to infants who have ongoing exposure to HIV-1 through breastmilk, can substantially reduce HIV-1 risk.[8] These infant studies provided compelling analogous evidence that antiretroviral prophylaxis could be highly efficacious for preventing infection in the context of known and ongoing HIV-1 exposure.[9] Evidence shows substantial reduction in HIV-1 quantity in the plasma and genital compartments of persons on suppressive ART, which translates into markedly reduced risk of HIV-1 transmission to sexual partners.[10] This evidence has informed the need to explore the use of ART for HIV prevention.

A meta-analysis conducted by Attia et al. showed significantly lower risk of HIV-1 transmission in heterosexual discordant couples whose HIV positive partner was on ART.[11] Similarly, the Partners in Prevention HSV/HIV Transmission Study conducted among 3,381 HIV-1 serodiscordant couples showed a 92% reduction (95% CI 43-100%, p=0.004) in HIV-1 transmission risk among the 349 couples in which the HIV-1 infected partners initiated ART during follow-up.[12]

More recently, the Partners PrEP Study, a phase III, placebo-controlled trial of oral tenofovir disoproxyl fumarate (TDF) and combination emtricitabine (FTC)/TDF PrEP among HIV-1 uninfected partners in 4758 HIV-1 serodiscordant African couples, demonstrated that PrEP substantially reduced HIV-1 risk. The study showed that HIV-1 protection from FTC/TDF and TDF was statistically similar (p=0.23), and each reduced HIV-1 in both men (TDF 63%, p=0.01 and FTC/TDF 84%, p<0.001) and women (TDF 71%, p=0.002 and FTC/TDF 66%, p=0.005).[13] The study result reinforced findings of other recent PrEP studies: in iPrEx, daily oral FTC/TDF reduced HIV-1 risk by 44% (95% CI 15-63%, p=0.005) among 2499 men who have sex with men,[14] and oral FTC/TDF reduced HIV-1 risk by 63% (95% CI 22-83%, p=0.01) among 1200 young heterosexuals from Botswana in the TDF2 study.[15]

In 2011, the observational data associating ART initiation with substantial reduction in HIV-1 risk were confirmed by HPTN 052, a randomized trial among 1763 HIV-1 serodiscordant couples.[16] Of 39 HIV-1 transmissions observed in the study, 28 were

---

8 Chasela CS, Hudgens MG, Jamieson DJ, Kayira D, Hosseinipour MC, Kourtis AP, et al. Maternal or infant antiretroviral drugs to reduce HIV-1 transmission. N Engl J Med 2010,362:2271-2281.

9 Mofenson LM. Protecting the next generation--eliminating perinatal HIV-1 infection. N Engl J Med 2010,362:2316-2318.

10 Wood E, Kerr T, Montaner JS. HIV treatment, injection drug use, and illicit drug policies. Lancet 2007,370:8-10.

11 Attia S, Egger M, Muller M, Zwahlen M, Low N. Sexual transmission of HIV according to viral load and antiretroviral therapy: systematic review and meta-analysis. AIDS 2009,23:1397-1404.

12 Donnell D, Baeten JM, Kiarie J, Thomas KK, Stevens W, Cohen CR, et al. Heterosexual HIV-1 transmission after initiation of antiretroviral therapy: a prospective cohort analysis. Lancet 2010,375:2092-2098.

13 Karim SS, Karim QA. Antiretroviral prophylaxis: a defining moment in HIV control. Lancet 2011,17:17; Cohen J. AIDS research. Complexity surrounds HIV prevention advances. Science 2011,333:393.

14 Grant RM, Lama JR, Anderson PL, McMahan V, Liu AY, Vargas L, et al. Preexposure chemoprophylaxis for HIV prevention in men who have sex with men. N Engl J Med 2010,363:2587-2599.

15 Thigpen MC, Kebaabetswe PM, Smith DK, Segolodi TM, Soud FA, Chillag K, et al. Daily oral antiretroviral use for the prevention of HIV infection in heterosexually active young adults in Botswana: results from the TDF2 study. In: 6th IAS Conference on HIV Pathogenesis, Treatment and Prevention. Rome, Italy; 2011. Abstract WELBC01.

16 Donnell D, Baeten JM, Kiarie J, Thomas KK, Stevens W, Cohen CR, et al. Heterosexual HIV-1 transmission after initiation of antiretroviral therapy: a prospective cohort analysis. Lancet 2010,375:2092-2098.

Approved Operational Copy

virologically-linked within the study partnership: 27 in the delayed ART arm and only 1 in the immediate ART arm, a 96% reduction in HIV-1 risk that was highly statistically significant (RR 0.04, 95% 0.01-0.27, p<0.001).[17]

Mathematical modeling by WHO has stimulated great interest in the potential of ART to substantially reduce population HIV-1 incidence when administered through near-universal annual HIV-1 testing, linkage to care, and uptake of ART, regardless of CD4 count (together called the 'Test and Treat' or 'Test and Linkage to Care' concept).[18] Most individuals are infected for several years before CD4 decline or clinical disease necessitates ART, and although WHO HIV-1 treatment guidelines now recommend ART initiation at CD4 counts <500 cells/μL, <200-250 remains the standard in many countries, and the average CD4 at ART initiation is <100 in many settings, often due to late testing or fears about ART.[19] While ART adherence has been excellent in Africa,[20] this success has been exclusively in individuals with advanced disease whose families are dedicated to provide tangible support to overcome severe structural and economic barriers to adherence because of the dramatic functional improvement they witness with ART. It is unclear whether asymptomatic individuals and their families will share the same commitment to adherence when ART is given to asymptomatic individuals.

1.2 Concern about PrEP and adherence

A case-cohort analysis of the Partners PrEP study alluded to the importance of adherence for the efficacy of PrEP. Among subjects on the active PrEP arms who acquired HIV-1 after randomization, 31% had tenofovir detected in a plasma sample at the seroconversion visit compared with 82% of a randomly-selected samples from a subset of subjects who did not acquire HIV-1, verifying overall high adherence in the trial and demonstrating that seroconverters had low adherence. Importantly, having detectable tenofovir was associated with a relative risk reduction for acquiring HIV-1 of 86% (TDF, p<0.001) and 90% (FTC/TDF, p=0.002) – further emphasizing high protection against HIV-1 for those who were PrEP-adherent.[21]

Across studies, adherence has been a key predictor of efficacy; very high adherence in the Partners PrEP Study likely explains the high degree of HIV-1 protection. Notably, two PrEP trials among African women – FEM-PrEP (using FTC/TDF) [37] and VOICE (using TDF and tenofovir gel),[22] failed to show HIV-1 protection. For the FEM-PrEP trial, substantial lack of adherence (approximately only 25% consistent use of the study medication) likely explains the failure to show HIV-1 protection. Also, the VOICE trial found that none of the PrEP products (oral and vaginal) were effective in preventing HIV after running for 3 years. The

17 Cohen MS, Chen YQ, McCauley M, Gamble T, Hosseinipour MC, Kumarasamy N, et al. Prevention of HIV-1 infection with early antiretroviral therapy. N Engl J Med 2011,365:493-505.

18 Granich RM, Gilks CF, Dye C, De Cock KM, Williams BG. Universal voluntary HIV testing with immediate antiretroviral therapy as a strategy for elimination of HIV transmission: a mathematical model. Lancet 2009,373:48-57.

19 Rapid advice: antiretroviral therapy for HIV infection in adults and adolescents. In: World Health Organization; 2009.

20 Ware NC, Idoko J, Kaaya S, Biraro IA, Wyatt MA, Agbaji O, et al. Explaining adherence success in sub-Saharan Africa: an ethnographic study. PLoS Med 2009,6:e11.

21 Extract from the Partners PreP Study Demonstration Project in Kenya protocol version 2.0

22 NIH modifies 'VOICE' HIV prevention study in women: oral tenofovir discontinued in clinical trial. In; 2011.

Approved Operational Copy

study also revealed that this finding was attributable to the poor adherence, since the drug was only detectable in less than a quarter of participants assigned to the treatment group.[23] More importantly when looking at the characteristics of the non-adherent participants, it appears that they were more risky groups, adherence was poorer among women under 25 years and among unmarried women. Thus, while a number of factors could explain the divergent trial results,[24] foremost being non-adherence[25]. Importantly, for HIV-1 uninfected members of known HIV-1 serodiscordant couples, as shown in the Partners PrEP Study, adherence appears to be very high.

Although perception of risk has been found to be a main predictor of health behaviours[26], only one study has focused on the effect of risk aversion on perceived risk of HIV and HIV status[27]. Further research is then needed in order to investigate the role of risk aversion on adherence to PrEP.

1.3 Use of ART for HIV-1 prevention in serodiscordant couples

A clear and consistent message from stakeholders has been the need to prioritize access of ART and PrEP for high risk populations. One such population is HIV-1 serodiscordant couples (i.e., one member is HIV-1 infected and the other uninfected). Population data from Africa suggest that a substantial fraction of new infections (up to half or more) may occur within stable serodiscordant marital or cohabiting relationships.[28] Epidemiologic studies, national HIV-1 serosurveys, and mathematical modeling analyses indicate that stable, heterosexual HIV-1 serodiscordant couples account for a substantial proportion of new HIV-1 transmissions in East Africa.[29]

Data from Nigeria also indicate that couples in stable relationships contribute significantly to HIV incidence. About 62% of new infections occur among persons perceived as practicing “low risk sex" in the general population, including married sexual partners (MOT study - NACA 2008).[30] Evidence suggests that the magnitude of serodiscordance among pregnant couples is high and is a significant channel for transmitting new HIV infections. In

[23] Marrazzo J, Ramjee G, Nair G, Palanee T, Mkhize B, et al. Pre-exposure Prophylaxis for HIV in Women: Daily Oral Tenofovir, Oral Tenofovir/Emtricitabine, or Vaginal Tenofovir Gel in the VOICE Study (MTN 003). 20th Conference on Retroviruses and Opportunistic Infections, Atlanta, GA, March 3–6, 26LB. 2013

24 Karim SS, Karim QA. Antiretroviral prophylaxis: a defining moment in HIV control. Lancet 2011,17:17.

25 Kashuba AD, Patterson KB, Dumond JB, Cohen MS. Pre-exposure prophylaxis for HIV prevention: how to predict success. Lancet 2011:6;6.

26 Anderson, LR., Mellor JM. Predicting health behaviors with an experimental measure of risk preference. Journal of health economics. 2008; 27(5): 1260-1274

27 Lammers J, Van Wijnbergen S. HIV/AIDS, Risk Aversion and Intertemporal Choice.2008

28 Dunkle KL, Stephenson R, Karita E, Chomba E, Kayitenkore K, Vwalika C, et al. New heterosexually transmitted HIV infections in married or cohabitating couples in urban Zambia and Rwanda: an analysis of survey and clinical data. Lancet 2008,371:2183-2191.

29 Dunkle KL, Stephenson R, Karita E, Chomba E, Kayitenkore K, Vwalika C, et al. New heterosexually transmitted HIV infections in married or cohabitating couples in urban Zambia and Rwanda: an analysis of survey and clinical data. Lancet 2008,371:2183-2191; Grabbe KL, Bunnell R. Reframing HIV prevention in sub-Saharan Africa using couple-centered approaches. JAMA 2010,304:346-347.

30 National Agency for the Control of AIDS. Modes of HIV transmission in Nigeria: analysis of the distribution of new infections in Nigeria and recommendations for prevention. 2010.

Approved Operational Copy

Nigeria, about 7.7% to 78.7% of HIV positive pregnant women who access antenatal care have HIV negative male sex partners. The highest rates occurred among married couples in Southern Nigeria.[31] Among the sero-discordant couples enrolled in the FHI 360 program, women were 11 times more likely to be the HIV positive partner than the HIV negative partner. [32]

Also, preliminary data from the IHVN project in Jos, Nigeria identified discordant couples as having elevated risk of acquiring HIV infection due to low condom use (<30%) between partners, the majority (>84%) of whom are married. IHVN records show 13% HIV seroprevalence in females and 7% in males in this group, much higher than the national average of 3.4%. Discordant couples make up about 3% of the total number of HIV positives in the IHV-N PEPFAR network.[33]

Understanding HIV-1 prevention choices and targeting prevention strategies to this group are public health priorities. In particular, HIV-1 serodiscordant couples have been specifically identified as a priority population for implementation of antiretrovial-based HIV-1 prevention, given their high risk, smaller number for targeting relative to the general population, ability to be targeted for prevention efforts through promotion of couples HIV-1 counseling and testing, and the clear advantage to the partnership in avoiding HIV-1 transmission. Importantly, both ART (HPTN 052) and PrEP (Partners PrEP Study[34]) have demonstrated high efficacy for HIV-1 protection when used by members of HIV-1 serodiscordant couples. WHO is poised to release guidelines for counseling and prevention for HIV-1 serodiscordant couples, which will include ART and PrEP as potential prevention strategies.

1.4 Staged PrEP until ART initiation for HIV-1 prevention in couples

Given the constrained resources for HIV-1 treatment and prevention, many questions need to be considered regarding the relative benefits of PrEP and ART for HIV-1 prevention. The Partners PrEP study constructed a mathematical model to examine the impact and cost-effectiveness of different strategies, including earlier initiation of ART and/or PrEP, for HIV-1 prevention for serodiscordant couples.[35] The analysis provided three main results. First, PrEP used prior to ART initiation can prevent infections in HIV-1 serodiscordant couples and, although the initial costs are high, they are substantially offset by reduced

Approved Operational Copy

---

31 Sagay AS, Onakewhor J, Galadanci H, Emuveyan EE. HIV status of partners of HIV positive pregnant women in different regions of Nigeria: matters arising. Afr J Med Med Sci. 2006 Dec;35 Suppl:125-9; Ikechebelu, J., S. U. Mbamara, et al. (2009). "Sexual practices of people living with HIV in South Eastern Nigeria." Niger J Clin Pract 12(4): 416-20; Khamofu H. Discordancy trends in Nigeria's PMTCT programme. Presentation at the consultative meeting on the roadmap for moving forward the agenda for PreP access in Nigeria. May 21-24, Abuja, Nigeria

32 Khamofu H. Discordancy trends in Nigeria's PMTCT programme. Presentation at the consultative meeting on the roadmap for moving forward the agenda for PreP access in Nigeria. May 21-24,<<What year?>> Abuja, Nigeria

33 Abimiku A, Garber G. Creating a common platform for HIV Vaccine Research and HIV care and treatment programme. The Nigeria Canadian AIDS Vaccine Project protocol.2010

34 Baeten JM, Celum C, Partners PrEP Study Team. Antiretroviral pre-exposure prophylaxis for HIV-1 prevention among heterosexual African men and women: the Partners PrEP Study In: 6th IAS Conference on HIV Pathogenesis, Treatment and Prevention. Rome, Italy; 17-20 July 2011.

35 Hallett TB, Baeten JM, Heffron R, Barnabas R, de Bruyn G, Cremin I, et al. Optimal uses of antiretrovirals for prevention in HIV-1 serodiscordant heterosexual couples in South Africa: a modelling study. PLoS Med 2011,8:e1001123.

future ART costs among HIV-1-uninfected partners who remain uninfected. Second, PrEP in serodiscordant couples could be as cost-effective as earlier initiation of ART (compared to existing practice) if PrEP has a sufficiently high effectiveness (>70%) and low cost of delivery. If used in couples that remain at high risk, PrEP could be as cost-effective as earlier ART. Third, in couples that remain at high risk, PrEP and ART could be used sequentially (PrEP in the uninfected individual prior to ART initiation for their HIV-1-infected partner) to deliver maximal benefit and best cost-effectiveness.

A similar preliminary result was found in a mathematical model that examined the impact and cost-effectiveness of strategies, including earlier initiation of ART or PrEP, for HIV-1 prevention for serodiscordant couples in Nigeria. The results of the modelling suggest that the best first intervention strategy for discordant couples in Nigeria would be to ensure that all HIV positives are offered ART at current national guidelines. Additional reduction in new infections could be achieved by promoting condom use amongst discordant couples, offering PrEP to HIV negatives until their partner initiates ART or giving HIV positive partners TasP. Also, additional survival gains could be achieved through condom promotion for couples and TasP for HIV positive partners, which would also be incrementally cost-effective. However the impact was highly dependent on people taking the drugs consistently over long periods of time[36].

Several factors affect the cost effectiveness of PrEP vs. ART for prevention in serodiscordant couples (Figure 1). Once a method of PrEP is chosen, costing it out for the uninfected partner is straightforward: the same drug at the same interval for a given period. PrEP in the form of one tablet per day, for example, represents a predictable cost per year.

Costing out ART is more complicated. After infection with HIV, the CD4 count of an infected person declines from a normal value of about 1000 cells / microliter until, absent clinical intervention, a diagnosis of AIDS is warranted at a count of 200. Under current guidelines, ART should be initiated at a count of 500 cells / microliter or below. After ART begins, the CD4 count begins to rise again and the viral load drops until, after about six months, the viral load is undetectable. After viral suppression, the risk of HIV transmission to the uninfected partner is greatly reduced.

The cost effectiveness question comes up if the infected person is the positive member of a serodiscordant couple. PrEP begins for the uninfected partner as soon as the infected partner is diagnosed and, since the uninfected partner is still uninfected, typically long before the CD4 count of the infected partner begins to decline. Once the infected partner presents an undetectable viral load, the risk of HIV transmission is greatly reduced. If this risk is less than or equal to the risk reduction provided by PrEP, then PrEP could be discontinued for the uninfected partner with no increase in risk. (The prevention strategy

36 Mitchell KM, Lépine A, Terris-Prestholt F, Vickerman P. Modelling the impact and cost-effectiveness of treatment as prevention and pre-exposure prophylaxis amongst HIV serodiscordant couples in Nigeria. *Pre-congress symposium on Program Science Perspective on STD/HIV Interventions, STI & AIDS World Congress, Vienna.*2013

.

Approved Operational

after this point is called "Treatment as Prevention" or TasP.) PrEP is, therefore, a continuous cost from the moment of the diagnosis of the infected parter until six months after the infected partner starts ART. Under current guidelines that is six months after the infected partner's CD4 count falls to 350.

In the chart (Figure 1), the vertical dashed line is set at about 75%, which was the efficacy of the FTC / TDF method of PrEP found in the Partner's PrEP study. If viral suppression after ART in the infected partner protects the uninfected partner at least this effectively, then PrEP can be discontinued in favor of TasP and the differential costs saved.

This raises a question. Once started, ART becomes a continuous lifetime cost. If the infected partner is going to start ART at some point anyway, could the same risk reduction as PrEP have been bought for this couple at less total cost by starting ART at a CD4 count greater than 350? In other words, is it more cost effective to start ART earlier, thereby moving to TasP earlier, and save the expense of PrEP over the shortened period of high risk to the uninfected partner?

The vertical axis (Figure 1) is the ratio of the total cost of PrEP to the total cost of ART for a couple. The ratio is expressed as a percent, so the dashed horizontal line at 100 marks the boundary where the costs of PrEP and ART are equal; above this line, total PrEP costs for a couple are less than total ART costs, below the line PrEP costs more than ART. Similarly, the dashed horizontal line at 50 marks the boundary where the total cost of PreP to a couple is half that of ART.

Approved Operational Copy

Figure 1: Cost effectiveness of long-term PrEP vs early ART

Total costs for PrEP and ART are computed within the context of a scenario. A scenario might be, for example, a stable higher-risk couple that becomes serodiscordant at age 30, computed through age 50. The value of the PrEP / ART ratio is based on the total costs for the

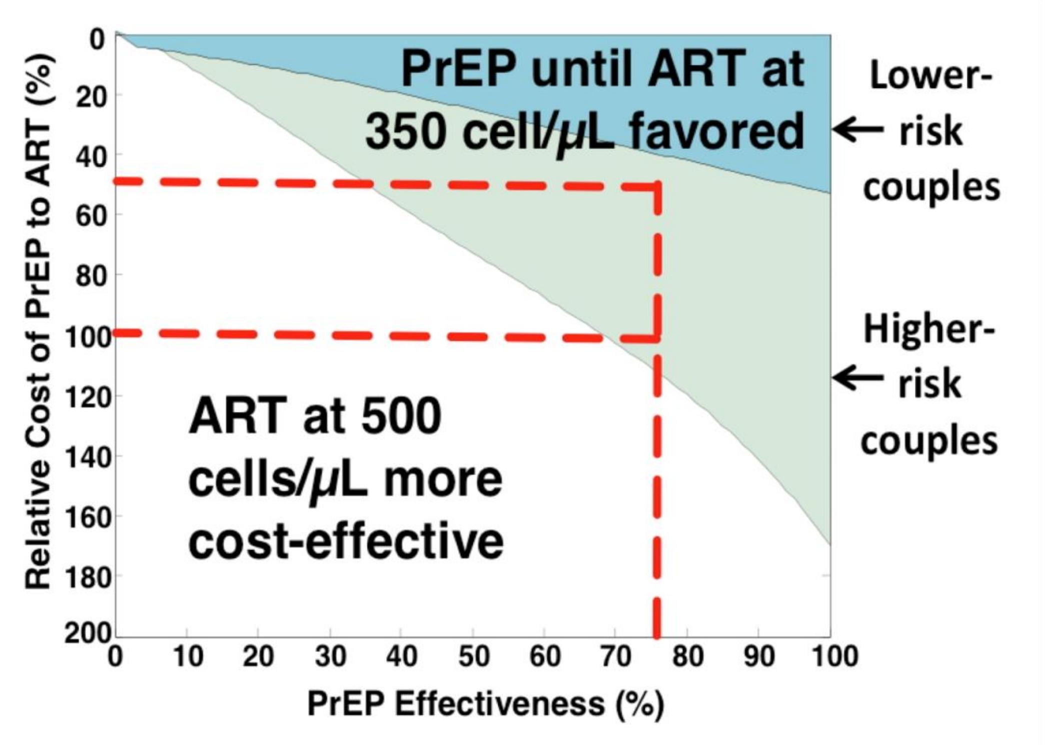

couple in this scenario, not just a range of prices for the drugs. If, for example, PrEP fails and the negative partner sero-converts and later goes on ART, then the ratio is found by taking the amount spent on PrEP for the partner who was originally uninfected divided by the amount spent on ART for both of them. Scenarios with higher ART costs (whether due to PrEP failure or to higher drug costs) will generate points higher up the cost ratio axis and favor starting ART later, at CD4 counts nearer 350. Similarly, scenarios with higher PrEP costs appear lower on that axis and favor starting ART earlier, nearer 500.

The shaded regions indicate the set of scenarios in which PrEP followed by ART at a count of 350 is at least as cost-effective as earlier initiation of ART (at CD4 <500 cells/μL) at allowing couples to be "alive and HIV-1 free at age 50." The dark shaded region corresponds to lower risk couples (heavily counseled clinical trial participants from the Partners in Prevention

HSV/HIV Transmission Study) and the lighter shaded region corresponds to higher-risk couples, which is the more typical behavior

The vertical dashed line represents the effectiveness estimate for FTC / TDF from the Partners PrEP Study of about 75%. It shows that, for higher-risk couples, PrEP used until

the HIV-1 infected partner starts ART at <350 cells/μL is cost-effective, even if the total cost of PrEP is as much as ART.

Ultimately, this result generated from the Partner's PrEP study showed that PrEP used prior to ART initiation can prevent infections in HIV-1 serodiscordant couples, with PrEP costs substantially offset by reduced future ART costs among HIV-1 uninfected partners who remain uninfected.

Importantly, ART and PrEP are not antagonistic -- staged and strategic use of PrEP and ART could substantially and cost-effectively reduce HIV-1 transmission in HIV-1 serodiscordant couples. As international and national guidelines regarding ART and PrEP use evolve (potentially to even earlier ART initiation), staged PrEP use would still be important to HIV-1 uninfected partners of HIV-1 infected persons not yet eligible for ART, not yet willing to start ART, and recently started on ART (i.e., during the first few months before viral suppression is attained). In the Partners PrEP Study, for example, where all HIV-1 infected partners did not meet national guidelines for ART initiation at the time of enrollment but were actively referred for therapy upon meeting national guidelines, 20% of HIV-1 infected partners started ART during a median of 23 months of follow-up.

1.5 The need for demonstration projects

The various PrEP studies have shown PrEP has the potential to have significant public health impact on the HIV epidemic. The next step is to evaluate how to best implement PrEP in different communities, including looking at the safety and effectiveness. Demonstration projects therefore represent the translation of research findings into practice. This would include demonstration of deliverability and understanding adherence and sexual behavior in the context of ART for prevention and PrEP use outside of clinical trials which are critical factors to consider in anticipating roll-out of these strategies. Also, the projects will assess the true realities associated with PrEP adoption, considering not only cost but also that it is only partially effective, requires regular HIV testing, ongoing medical monitoring, and has possible side effects and toxicities.

The first of many demonstration projects is the iPrEx open label extension study conducted in 11 sites in USA, Brazil, Peru and Ecuador. It is designed to provide additional information about the safety of PrEP and the behavior of people taking PrEP over a longer term. The study was opened to past study participants and enrolled for 12 months. The study hypothesis is that participants' knowledge that PrEP provides some protection against HIV infection, and that all study participants are receiving PrEP and not a placebo, will lead to increased use of the study drug, and increased protection against HIV infection.[37]

The San Francisco Department of Public Health also launched a demonstration project to evaluate the delivery of pre-exposure prophylaxis (PrEP) in STD clinics. The program will enroll up to 300 HIV-uninfected men who have sex with men (MSM) and transgender women at the San Francisco City Clinic and an additional 200 participants at a Florida

Approved Operational Copy

37 What is IPrEx OLE. http://www.iprexole.com/1pages/aboutus/aboutus-whatisiprexole.php Accessed 5th November, 2011.

Department of Health STD clinic in Miami, Florida.[38] There are planned PrEP demonstrations projects in other parts of USA, Kenya and South Africa also.

The successful result of the HPTN 052 study and the recently released WHO ART guideline (which recommends commencing ART at a CD4 count of 500 instead of the previous recommendation of 350), which would promote access of HIV discordant couples to ART irrespective of CD4 count, also calls for careful guidance and planning especially for resource-constrained countries like Nigeria. Demonstration projects can enable a country to plan for cost effective roll out programs, thereby ensuring maximal benefit from invested resources.

HIV-1 serodiscordant couples are a primary target for implementation of ART for prevention (TasP) and PrEP. Critical unanswered questions for successful implementation of antiretroviral-based HIV-1 prevention include: how to target these expensive prevention strategies to realize maximum population HIV-1 prevention benefits; whether HIV-1 infected persons with asymptomatic disease would accept ART to reduce their risk for transmitting HIV-1; whether at-risk HIV-1 negative persons would use PrEP, and whether couples would sustain the high adherence needed for high effectiveness. Couples may potentially have the opportunity to use ART or PrEP for prevention; however, costs would prohibit simultaneous use in most settings. Thus, staged use – i.e., PrEP until the HIV-1 infected partner initiates ART and achieves viral suppression – may be an effective and cost-effective approach,[39] and it is the approach we propose.

Successful implementation of ART and PrEP for prevention in HIV-1 serodiscordant couples will need to 1) target delivery to highest-risk couples, 2) respond to couples' preferences for and barriers to use of PrEP and ART, 3) achieve high uptake and sustained adherence and 4) consider how to discontinue PrEP if the HIV-1 infected partner initiates ART. This study proposes to address these key questions. In addition, for resource-poor settings like Nigeria, such PrEP demonstration projects must be able to show clearly models for effective target delivery of PrEP in a way that can ensure access and scale up of PrEP access by those who are at higher risk of contracting HIV infection beyond this initial target population thereby positively impact the National HIV epidemic.

Translating these findings into health service programs also poses many challenges that can be exacerbated in the context of weak health care delivery systems. This was evident with the current nevirapine-based programs for the prevention of mother-to-child transmission (PMTCT) in Nigeria. Despite the efficacy of the PMTCT program, only about 20.2% of HIV positive pregnant women have access to it in Nigeria.[40] This demonstration study is an opportunity to answer critically important implementation questions about how best to incorporate PrEP into routine health services for serodiscordant couples and how to make it accessible to HIV negative men and women in serodiscordant relationships

38 Press Release. SFDPH to Launch PrEP Demonstration Project for HIV Prevention. http://www.iprexnews.com/content/whatisnew.html. Accessed 5th November, 2012

39 Hallett TB, Baeten JM, Heffron R, Barnabas R, de Bruyn G, Cremin I, et al. Optimal uses of antiretrovirals for prevention in HIV-1 serodiscordant heterosexual couples in South Africa: a modelling study. PLoS Med 2011,8:e1001123.

40 National Agency for the control of AIDS. Draft 2012 Mid-term review of the 2010=2015 National HIV/AIDS strategic plan. 2013

Approved Operational Copy

who would benefit most from this product. This study will enable us understand how to prepare for widespread roll-out access following licensure of Truvada for use for HIV prevention in Nigeria especially with respect to providing adequate adherence support and safety monitoring in users. This is important as population-level effectiveness of PrEP may drop significantly without addressing our understanding of how to address potential challenges to product use and access including how to ensure adequate product supplies at health care delivery sites. A good understanding of the current health system delivery strengths and challenges prior to roll-out of new interventions is key to success.

1.6 Public Opinion about the use of PrEP in Nigeria[41]

As part of the plans for the implementation of the PrEP demonstration project in Nigeria, a feasibility study was undertaken. The feasibility study was designed as qualitative exploratory study with a purposive qualitative sample of key informants representing multiple perspectives on the broad question of how to roll out PrEP in a practical demonstration in Nigeria. Public opinion was sought on population to be targeted for PrEP, appropriate public communication strategies for PrEP, community engagement and partnership plans, and how to address administrative and logistic challenges with PrEP implementation in Nigeria. The study conducted in-depth interviews, focus group discussions, telephone interviews, online surveys and consultative meetings with various and multiple stakeholders in Nigeria.

The outcome of this extensive community consultation process showed that the Nigerian public were interested in and supportive of the use of PrEP as a HIV prevention measure as an addition to other HIV prevention strategies in the country. Overall, sero-discordant couples were identified as the priority target population for PrEP in the pilot study. However, the likely challenges with use of PrEP include having healthy persons take drugs and visit the health facilities regularly when not ill, giving blood samples, fear of creating drug resistance and the future sustainability of the program. In addition, stigma was a potential challenge for the use of ARVs by HIV negative individuals and a potential challenge for recruitment of HIV negatives into the study.

PrEP delivery services need to be integrated into existing ART structures/services and the prescription and dispensing of daily drugs should be handled by health professionals. Community engagements and extensive public enlightenment programmes through partnership with various agencies and organizations, especially those already involved in HIV prevention measures was highly recommended.

1.7 Potential impact of PrEP for HIV prevention in Nigeria

41 The Georgetown University supported NACA to conduct a feasibility study. The feasibility study was conducted in phases. The first phase involved the collection of data on the field from various stakeholders using both in-depth interviews and focus group discussions. Key informants included policy makers, development partners, researchers, health workers engaged with HIV treatment and prevention, academia engaged with HIV research, ethicists and members of HRECs, community representatives, HIV sero-discordant couples, men who have sex with men, male and female sex workers, injecting drug users, and representatives of women organizations, journalists, religious leaders. Respondents were drawn from mostly urban areas in Benue, Edo and Cross River, Abuja and Lagos. Two hundred and twenty one persons were directly reached through this process. The field work was complemented with telephone interviews with 131 persons, and an online survey to which 65 persons responded. The telephone and online survey explored similar themes to the field study.

Approved Operational Copy

As a preliminary effort to planning a PrEP demonstration project, it was important to understand the potential impact of the use of PrEP on the HIV epidemic in Nigeria, including the cost effectiveness of running such a programme for sero-discordant couples. The outcome of the model showed that that 40% of any projected reduction in HIV incidence would be due to expanding ART access over current levels to reach all people with CD4 ≤350, in accordance with national guidelines. Adding TasP, long-term PrEP and condom promotion was projected to avert the most infections; condom promotion was projected to be the most cost-effective per infection averted. The best first intervention strategy for discordant couples in Nigeria would be to effectively implement initiation of ART at CD4 ≤350 for all persons who are eligible for enrolment into the national treatment programme. As resources become available, additional reduction in new infections could be achieved by adding short-term PrEP (the use of antiretroviral by the HIV negative partner until the HIV positive partner has started ART, after which the HIV negative partner discontinues PrEP), and TasP for serodiscordant couples. The addition of long-term PrEP to the mix was not predicted to be cost-effective. The societal cost of implementing condom promotion programmes makes condom promotion not a cost effective programme for reducing HIV incidence in the long term.[42]

1.8 Planning for PrEP administration and logistics in Nigeria

As part of the plans for the preliminary plans for the conduct of the PrEP demonstration project, consultative meetings were held with representatives of sero-discordant couples and policy makers.

The first meeting was with nine sero-discordant couples resident in the states where the PrEP demonstration project was initially proposed to hold - Cross River, Benue, Abuja and Edo states. In addition, there were four representatives from national civil society organizations who themselves are living with HIV. The meeting helped to identify barriers and facilitators to implementing PrEP with sero-discordant couples; identify mechanism to address the barriers; and develop strategies for recruiting, educating and ensuring access and uptake of PrEP by serodiscordant couples. The second meeting was with policy makers with the aim of identifying how to address the administration and logistics challenges that may be faced with the implementation of PrEP. The outcome of the meetings was a consensus on the implementation model(s) for the PrEP demonstration project.

The consultative meetings highlighted the need to address adherence support since it would be challenging for HIV negative individuals to continue to take pills and visit hospitals. Also health care providers would need to be retrained on how to ensure client confidentiality as this is a deterrent for hospital visits by any individual. Community training on stigma reduction was also important so as to improve community understanding of PrEP and community support. The creation of support group for the sero-discordant couples was seen as important and critical for the success of the

42 Idoko J. Feasibility of Implementing Biomedical Prevention Program in Africa: The case study of Nigeria. Presented on the 19th of November 2013 at the 2013 Biomedical HIV Prevention Forum, Abuja, Nigeria. http://nhvmas-ng.org/forum/slides/Feasibility%20of%20Implementing%20Biomedical%20Prevention%20Program%20in%20Africa_%20The%20case%20study%20of%20Nigeria%20-%20John%20Idoko.pdf

programme as this would encourage recruitment of HIV negative females with HIV positive male partners. Recruitment of HIV positive males can also be improved through the use of community-based testing including the use of incentive based testing. Testing should target migrant or mobile work forces like miners and bankers. Care givers who can identify and support couples within the communities for the PrEP program should also be engaged for the programme. It was also important that the programme ensures that drugs and test kits were continuously available at facilities as these are major deterrants to adherence.

In each of the demonstration project States, secondary health facilities should serve as the recruitment and central service delivery point for PrEP clients, while the primary health centers and private hospitals in the state should be engaged to provide drug refills. Recruitment of clients from STI and TB clinics was considered important. Male involvement in the PrEP demonstration project was also viewed as critical. PrEP services should be centralize to the coordinating secondary facility for the first three months which is a critical period in any care program. All data collection process should be centralized. Also, six-monthly laboratory tests to evaluate clients' health profile should be centralized to coordinating secondary health facilities. Drug refills and the 3 monthly HCT should be decentralized to private hospitals and primary care centers. Community engagement programmes activities should also be decentralized. Training on adherence counseling and quality health care service provision needs to be conducted consistently at both centralized and decentralized sites.

Based on the outcome of the deliberations, it was concluded that:

1. In Cross River State, PrEP service should be integrated into the general out-patient health care delivery service structure
2. In Edo State, PrEP service should be centralized to the secondary health care facility
3. In Benue/Plateau State, PrEP service should be decentralized, with central services provided at the secondary health care facility and decentralized services offered at the primary health care facilities.

1.9 PrEP as part of a complete prevention package

While the efficacy of PrEP in reducing the risk of HIV infection has been established, PrEP would be provided to serodiscordant couples participating in the study, along with multiple HIV prevention tools that will address both behavioral and biomedical HIV prevention needs. For populations at risk for HIV infection, a comprehensive HIV prevention package would include regular HIV testing, access to condoms, counseling on condom use and risk reduction, STI testing and treatment, and linkage to other prevention services. Participants will also receive counseling support for pill-taking to encourage adherence and blood tests to monitor for safety. It would be expected that all patients who plan to be on PrEP should also take a complete dose of hepatitis B vaccination.

Approved Operational Copy

1.10 The Quality Improvement (QI) approach to service delivery

This study will adopt the Quality Improvement approach as used in the CAPRISA 008 study. This is in view of the need to strengthen the health care delivery system needed to support the implementation of this demonstration project.

The QI approach is based on methodology that is grounded in operations research and management science, two well-established fields that have, for more than 90 years, combined the disciplines of statistics, psychology, systems engineering, and iterative learning, to have a major impact on systems performance across countries and industries. This approach seeks to design systems for maximum effectiveness, efficiency, and adaptability and to actively disseminate the best models for health service delivery at the most rapid rate possible. Specialized, evidenced-based tools aimed at rapid-cycle iterative testing of changes, networked collaborative learning, development of institutional capability for continuous improvement and frameworks to guide large-scale change have been developed to facilitate this process. The QI approach supports a shift in provider attitudes and practice from a prescriptive mode to one that supports critical thinking and problem solving skills with continuous review and improvement of service provision.

Health care quality improvement principles and the "Model for Improvement" provide an effective approach to help close the gap between evidence-based knowledge and the ability of health systems to implement large-scale programs. This approach places a premium on data driven front line decision making, peer-to-peer knowledge exchange, local adaptation of clinical protocols, and highly participative management. Originally developed in the United States and widely adopted in the United Kingdom and other high-income nations, these efforts have increasingly found their way, with appropriate modifications, into global health applications in low and middle income countries.

Application of the QI approach to health systems of low and middle income countries shows considerable promise. Efforts in the Russian federation have reduced neonatal mortality by 60%;[43] in Niger, malnutrition related fatality was halved in a single year;[44] in Ecuador, an essential obstetric care collaboration substantially reduced the incidence of post-partum hemorrhage;[45] in projects in South Africa, rapid scale-up of access to HIV care and treatment services and falling rates of mother-to-child transmission of HIV at a district level have been demonstrated.

Traditionally this approach is used following policy formulation and where some program implementation experience has already been established. This often means that health care providers have to unlearn what they have been doing for years and replace it with new enabling and empowering approaches that involve critical thinking and problem solving skills, which have the added advantage of application to other health challenges facing the

43 Quality Assurance Project. Improving the System of Care for Neonates Suffering from Respiratory Distress Syndrome in Tver Oblast. Bethesda, MD: University Research Corporation; 2001.

44 Catsambas TT FL, Gutmann M, Knebel E, Hill P, Lin Ya-Shin. . Evaluating Health Care Collaboratives: The Experiences of the Quality Assurance Project. . Bethesda, MD: University Research Corporation;2008.

45 Hermida J RM, Vaca L, Ayabaca P, Romero P, Vieira L. . Scaling Up and Institutionalizing Continuous Quality Improvement in the Free Maternity and Child Care Program in Ecuador: Latin America and Caribbean Regional Health Sector Reform Initiative Report. . Bethesda, MD: University Research Co LLC; 2005.

Approved Operational Copy

facility or service. Like the CAPRISA 008, this demonstration project will be testing this model in a rigorous manner through strengthening existing out-patient clinics and HIV service delivery clinics as a foundation to introduce a new health technology even prior to licensure and importantly pave the way for more rapid, efficient, safe and effective access to a much needed product. A strengthened service delivery point and more engaged providers will benefit existing service centres, overall health care delivery in these facilities and most importantly support and benefit the clients of these services.

1.11 Risk aversion as a determinant of adherence to PrEP among serodiscordant couples

This project will also be studying the potential role of risk aversion as a factor that could influence PrEP adherence among serodiscordant couples. In view of the critical role of adherence in the successful implementation of PrEP programmes, it would be important to identify the main drivers of adherence to PrEP, identify the possible role of risk aversion on adherence to PrEP, and identify how measures to address risk aversion may otherwise influence PrEP adherence in this project.

Risk aversion is measured by looking at how people behave when they are exposed to risk or uncertainty. People who are risk averse will try to avoid risks while risk lovers will take risks. Psychologists have developed scales in order to measure attitudes toward risk in different domains. These include the Rohrmann scale[46] which assesses attitudes toward different hazard types such as physical/accident, physical/illness, financial and social hazards, and the risk taking Index of Nicholson et al[47] which includes several domains such as recreational, health, career, finance, safety, social. The Nicholson et al. scale for instance shows that for health the risk index score is 4.62 on average while it is 4.14 for finance. For women, the difference is even greater (4.31 versus 3.65). If we look at correlations between the health and finance domain, Nicholson et al. paper find a correlation of 0.21 and Rorhmann a correlation around 0.4 Dohmen et al[48] however, showed evidence that each specific domain has a higher predictive power of related behaviours, so that risk aversion in finance performs better to predict investment in stocks while risk aversion in health has a stronger power to predict smoking. This shows the importance of having a measure of risk aversion that is closer to the domain of the outcome analysed.

Economists have used experimental games by exposing participant to some uncertainty or risk in order to measure their risk aversion. Games were developed partly because of the difficulty to measure behavior-like risk aversion, and the difficulty to obtain exogenous measures (that are not correlated with other characteristics of the person) by directly asking subjective questions to subjects as done by psychologists. There are two

---

46 Rohrmann B. Risk attitude scales: concepts, questionnaires, utilizations. Project report. 2005. Online access http://www. rohrmannresearch. net/pdfs/rohrmann-ras-report. pdf, 13, 2012

47 Nicholson N, Soane E, Fenton-O'Creevy M, Willman P. Personality and domain-specific risk taking. Journal of Risk Research. 2005; 8(2): 157-176

48 Dohmen T, Falk A, Huffman D, Sunde U, Schupp J, Wagner GG. (2011). Individual risk attitudes: Measurement, determinants, and behavioral consequences. Journal of the European Economic Association. 2011; 9(3): 522-550

categories of games: elicitation method versus multiple price list method[49] summarized in Table 1 below. From the review by Charness et al, it appears that although the balloon analogue risk task (BART) may predict successfully risky behaviours associated with HIV, it will not be easy to implement as it requires a lab with computers. The Holt & Laury game has been the method most widely used in the literature although in our study we believe that the Eckel & Grossman could also be of a strong interest.

The Holt & Laury game consists in asking participants to choose between a series of 10 paired gambles where Gamble B is a more-risky gamble than Gamble A. The game is designed so that most participants will start by choosing Gamble A and will end by choosing Gamble B. The risk aversion is computed by using the switch point at which participant decides to go for the paired risky gamble (Gamble B). The game is designed so that a risk neutral will switch at the gamble 4, those switching before are risk lovers and those switching later to the risky gamble are risk adverse. Under the assumption of constant relative risk aversion (CRRA), utility can be represented by this function:

$U(X)=\frac{x^{1-r}}{1-r}$ and where r=0 if risk neutral, r>0 if risk averse and r<0 if risk seeker

Figure 2: Holt & Laury game

Option A	Option B	Expected payoff difference
1/10 of $2.00, 9/10 of $1.60	1/10 of $3.85, 9/10 of $0.10	$1.17
2/10 of $2.00, 8/10 of $1.60	2/10 of $3.85, 8/10 of $0.10	$0.83
3/10 of $2.00, 7/10 of $1.60	3/10 of $3.85, 7/10 of $0.10	$0.50
4/10 of $2.00, 6/10 of $1.60	4/10 of $3.85, 6/10 of $0.10	$0.16
5/10 of $2.00, 5/10 of $1.60	5/10 of $3.85, 5/10 of $0.10	−$0.18
6/10 of $2.00, 4/10 of $1.60	6/10 of $3.85, 4/10 of $0.10	−$0.51
7/10 of $2.00, 3/10 of $1.60	7/10 of $3.85, 3/10 of $0.10	−$0.85
8/10 of $2.00, 2/10 of $1.60	8/10 of $3.85, 2/10 of $0.10	−$1.18
9/10 of $2.00, 1/10 of $1.60	9/10 of $3.85, 1/10 of $0.10	−$1.52
10/10 of $2.00, 0/10 of $1.60	10/10 of $3.85, 0/10 of $0.10	−$1.85

Approved Original Copy

49 Charness, G., Gneezy, U., & Imas, A. (2013). Experimental methods: Eliciting risk preferences. Journal of Economic Behavior & Organization, 87(0), 43-51. doi: http://dx.doi.org/10.1016/j.jebo.2012.12.023

Table 1: Methods to elicit risk preferences

Method	Name	Description	Advantages	Disadvantages
Elicitation	The balloon analogue risk task (BART)	Pump air in a balloon, each pumps leads to earn money but if the balloon pops you lose everything	Design to model situations where excessive risk taking leads to diminishing returns and greater hazards, found correlated to certain risk behaviours such as gambling, drug use and unprotected sex	Not clear if extend to other domains, require a computer and multiple trials to implement, may not be adapted when time is a factor and where access to computers is limited
	Questionnaires	Rate your willingness to take risks in general on a 10 point scale	Easy to implement	Assumes risk constant across domains while risk seems to differ between domains<br>No incentive in the questionnaire then not sure it reflects true attitude towards risk
	Gneezy and potters	The decision maker receives $X and is asked to choose how much will be invested in a risky option and how much will be kept	Good to predict risk preferences in the context of financial decision-making, and to compare gender differences in risk attitudes	Cannot distinguish between risk seeking and risk neutral
	Eckel and grossman	Chose 1 gamble in a list of 6 gambles	Easy to understand	None listed
Multiple price list (MPL) method	Holt & Laury: choice between gamble as multiple price list	List of 10 decision between paired gambles (gamble A is a safe gamble and	Widely used so possible to make comparison between different context	Hard to understand can result to inconsistent choice

Approved Operational Copy

		gamble B a risky gamble) , switch point is used to measure individual's risk preference		

The chosen gamble implies an interval for the risk coefficient that corresponds to the switch point; this interval is determined by calculating the value of r that would make the individual indifferent between the gamble chosen and the two adjacent gambles. For example a choice of gamble 3 implies a risk coefficient of (-.0.49; -0.15) where indifference between Gamble 2 and 3 corresponds to r=-0.49 and indifference between Gamble 3 and 4 corresponds to r=-0.15.

Two approaches have been adopted when using the Holt & Laury game. Firstly, hypothetical financial outcomes i.e. where individuals were asked to choose between 10 paired hypothetical risky lotteries. Secondly, in real payment experiments, participants are asked to do the same but at the end of the experiment one of their choice is randomly selected and is played for real and participants earn the outcome of the lottery. There is some evidence to suggest that people react differently hypothetical and real payment as we observe that individuals are more risk averse with real payment.[50]

Application of the Holt & Laury experiment suggests that most individuals are risk averse, with around less than 10% being risk-lovers i.e. with a negative risk aversion ratio. This experiment then allows having a continuous measure of risk aversion providing information on the degree of risk aversion.

There is some limitation when using the Holt & Laury experiment. The game is difficult to understand so that the game can result in inconsistencies due to the poor understanding of the game by participants.[51] Moreover, the game requires to some extent a good understanding of probabilities and may not be easy to implement among uneducated participants. For these reasons, it is important to also use the Eckel & Grossman elicit risk aversion.[52] This game also requires players to choose between less risky and more risky lottery. However, the game only just involves one task that consists of choosing one lottery among a set of six lotteries. Conversely to the Holt & Laury game, this game assumes a similar probability of occurrence of the high and low payment in each lottery so that it does not require a good understanding of probabilities.

For the PrEP demonstration project, the Eckel & Grossman games which measures monetary outcomes into health outcomes will be adapted. Instead of Naira, the outcome

50 Holt CA, Laury SK. Risk aversion and incentive effects. American Economic Review. 2002; 92(5): 1644-1655

51 Dave C, Eckel CC, Johnson CA, Rojas C. Eliciting risk preferences: When is simple better? Journal of Risk and Uncertainty, 2010; 41(3): 219-243; Jacobson S, Petrie R. Learning from mistakes: What do inconsistent choices over risk tell us? Journal of Risk and Uncertainty. 2009; 38(2): 143-158

52 Eckel CC, Grossman PJ. Men, women and risk aversion: Experimental evidence. Handbook of experimental economics results. 2008;1:1061-1073

Approved Operational Copy

will be the number of days in perfect health. This game is based on the assumption that individuals are not perfectly satiated in their level of time in full health. The adaptation of the game requires converting one unit of money in one day in full health, and use this measure to measure risk aversion in health. This will allow recovering the impact of income controlling for risk aversion. Empirical evidence suggests that wealthier households invest in more risky productive activities and higher returns.[53] This means that income is expected to be negatively correlated with risk aversion and that its effect on adherence if we do not control for risk aversion is likely to be underestimated, justifying the finding that income status is not always correlated with adherence (Falagas, Zarkadoulia, Pliatsika, & Panos, 2008).[54]

1.12. Measure of Adherence

There are several methods to measure adherence going from the most subjective measures (self-reported adherence) to the most objective measures (biological markers). A review of the different measures of adherence to antiretroviral medications has been developed by Miller and Hays[55] and includes self-reported adherence, clinician estimated adherence, diaries, pharmacy record, clinic attendance, plasma level and electronic monitors. Each measure presents some advantages and disadvantages, even the most objective one that consists in measuring the level of drug in blood sample. In fact, a main limitation of plasma level being that participants who are aware that adherence will be assessed will be more likely to use the product just before the adherence is being assessed. Evidence from the VOICE trial however shows that participants reported extremely high self-reported adherence (about 90%) but adherence assessed through plasma level was only 25% for vaginal PrEP and 30% for oral PrEP, justifying that plasma level may provide in the context of PrEP an unbiased measure of adherence. Another method for assessing adherence is through the use of the Medication Event Monitoring System (MEMS) Track caps, an electronic monitoring method. The caps have a microelectronic circuit that registers the exact dates and times of all bottle openings, thus providing more detailed information about the timing of doses than can be obtained through most other methods.

2.0 Study Method

This is a demonstration project to evaluate the effectiveness of three models for the delivery of PrEP and TasP as part of a combination prevention strategy for serodiscordant couples in three states in Nigeria. The overall goal is to identify the best model to facilitate uptake of PrEP as part of a comprehensive HIV prevention package for the HIV negative partner in serodiscordant relationships whose HIV positive partner has not begun ART or

53 Rosenzweig MR., Binswanger HP. *Wealth, weather risk, and the composition and profitability of agricultural investments* (Vol. 1055): World Bank Publications. 1992; Shaw KL. An empirical analysis of risk aversion and income growth. Journal of Labor Economics. 1996; 626-653

54 Falagas ME, Zarkadoulia EA, Pliatsika PA, Panos G. Socioeconomic status (SES) as a determinant of adherence to treatment in HIV infected patients: a systematic review of the literature. Retrovirology.2008; *5*(1): 1-12.

55 Miller L, Hays R. Measuring adherence to antiretroviral medications in clinical trials. HIV Clinical Trials. 2000; 1(1): 36-46

Approved Operational Copy

has been on ART for less than six months. PrEP (daily oral FTC / TDF) will be offered to the HIV-1 uninfected partner. Uptake of PrEP and TasP and adherence to PrEP will be measured. Factors (individual, facility and community level) that facilitate PrEP and TasP uptake and adherence to PrEP will also be assessed.

HIV-1 infected participants will be offered the opportunity to use ART support services regardless of their CD4 count. This is in line with the current national guidelines on HIV treatment for adults and adolescents (2010:13). The current guidelines enable HIV positive individuals in discordant relationships to have access to ART irrespective of the CD4 count. This project will therefore serve as a measure of the effectiveness of this program in Nigeria.

For each couple, if the HIV-1 infected partner initiates ART, we will discontinue PrEP for the HIV-1 uninfected partner six months later (once viral suppression is typically achieved). If the HIV-1 infected partner declines to initiate ART, or initiates and discontinues, we will continue PrEP. Mixed-methods work will be conducted to understand user preferences, couples decision-making, and barriers to uptake of and adherence to both PrEP and ART.

In Cross River State, PrEP will be provided through the outpatients department. The model will evaluate the effectiveness of integration of PrEP service delivery using an integrated approach for access and uptake of both PrEP and TasP services for both HIV negative and the index partners.

In Plateau State, PrEP and TasP will be provided through a decentralized service delivery model. PrEP related service will be obtained from a secondary health facility for the first three months as this is considered a critical period in any care program. Thereafter, services would be decentralized to primary and private project satellite clinics (primary and private centres). The satellite centres will provide the three monthly HCT services and monthly drug refill services. Six monthly laboratory assessment of health profiles would however, be conducted at the central hospital.

In Anambra State however, all PrEP and TasP services will be provided through HIV treatment centres with services remaining centralized.

3.0 Objectives

3.1 Primary objectives

3.1.1 Optimize, implement and evaluate the effectiveness of an implementation model which integrates PrEP and TasP provision into existing service provision centres.

3.1.2 Estimate the cost and cost effectiveness of the PrEP and TasP demonstration project.

3.1.3 Model the resources needed to provide scaled up PrEP and TasP access for serodiscordant couples in Nigeria.

Approved Operational Copy

Objective 3.1.1: To optimize, implement and evaluate the effectiveness of an implementation model which integrates PrEP and TasP provision into existing service provision centres.

The demonstration project will be evaluating the effectiveness of a model of HIV service delivery to couples that promotes and ensures effective PrEP access. Such a model should be able to ensure effective community engagement in ways that improve and support a couple's access and uptake of HIV prevention and treatment interventions, ensure access in hard to reach areas where there are few health facilities, and support sustained quality and consistency of PrEP and TasP services irrespective of place of delivery. The model for delivery of ART based HIV intervention programs for couples for this demonstration project would be designed based on feedback received during the formative research conducted to support this research. The study outcome should help critical stakeholders know how well the study model was able to achieve the desired PrEP service delivery into family planning services. This will help inform decision making on how to institutionalize the program, strategies that are economical and efficient, and how best to make modifications to the program implementation model to increase effectiveness.

The primary outcome of this objective is the measure of the impact that project intervention had on serodiscordant couples' access, uptake and retention in the antiretroviral-based HIV-1 prevention program. Formative and summative evaluations would be conducted. Formative evaluation will be conducted at months 6, 12 and 18 with derived information use to modify the program so as to increase its efficiency. The summative evaluation will be conducted at the end of the project and would help to decide on the effectiveness of the program.

The primary measure of this objective will be the number of new infections averted by the use of PrEP and TasP.

Objective 3.1.2: Estimate the cost and cost effectiveness the PrEP and TasP demonstration project.

A critical aspect of implementation is to obtain estimates of the cost of implementing PrEP on a small scale. Incremental financial and economic costs of provision will be collected from the provider's perspective for the duration of the demonstration project. Financial costs represent actual project expenditures, while economic costs represent the full value of all resources used in the intervention. Effectiveness will be modelled based on the Nigerian epidemic and intermediate demonstration trial outcomes to provide life years gained and infections averted. These will be matched with costs to estimate the cost effectiveness of the intervention in terms of cost per life year gained and cost per infection averted. Patient travel and opportunity costs will also be collected. This will provide insights into the travel costs incurred by the project to support transportation of study participants from satellite sites to the main study sites as well as other costs of participation

The primary measure of this objective is cost per life year gained and cost per infection averted.

Objective 3.1.3: Model the resources needed to provide scaled up PrEP and TasP access for serodiscordant couples in Nigeria.

While objective 3.1.2 will provide insights into the cost-effectiveness at demonstration project scale and target population level, this objective will enable the team make projections on what the real cost of scaling up the project will be. We will model the cost of a national PrEP program targeting serodiscordant couples. Using the cost data generated during the demonstration project, a cost model will estimate the total financial resources needed to scale up to a regional or national program as deemed appropriate. Further analysis will be undertaken to explore non-financial constraints such as personnel and infrastructural constraints.

The primary measure of this objective is an estimate of the total budget necessary to scale up PrEP and TasP nationally within the various target groups.

3.2 Secondary objectives

3.2.1 Assess client, facility and community level factors influencing demand, preferences, uptake and adherence for antiretroviral-based HIV-1 prevention in Nigeria

3.2.2 Assess user preferences among high-risk HIV-1 serodiscordant couples for ART initiation for HIV-1 infected partners and PrEP for HIV-1 uninfected partners.

3.2.3 Ascertain initiation of and adherence to PrEP among HIV-1 uninfected partners as a bridge to ART.

3.2.4 Look for evidence of risk compensation by HIV-1 serodiscordant couples on PrEP or TasP, migration from condom use.

3.2.5 Determine the ART resistance profile developed by HIV-1 serodiscordant couples on PrEP during the study; assess that profile's prevalence.

3.2.6 Model the resources needed to provide PrEP and TasP for other specific target groups who are also at high risk of contracting HIV.

Objective 3.2.1: Assess client, facility and community level factors influencing preferences, uptake and adherence for antiretroviral-based HIV-1 prevention in Nigeria

Understanding factors that influence couples' preferences and their likelihood to initiate and adhere to PrEP and/or ART in a real-world setting is critical for providers who will counsel HIV-1 serodiscordant couples.

Using interviewer-administered questionnaires, assessments will be made of the correlates to preferences, uptake and adherence to ART and PrEP. These will include characteristics of sexual risk (e.g., sexual frequency, condom use, whether the HIV-1 uninfected partner reports outside partners); depression and substance use; gender of the HIV-1 uninfected partner; fertility intentions; partnership characteristics (whether the couple is cohabiting at baseline, has children, is together throughout follow-up, whether members have other partners, and whether the HIV-1 infected partner initiates ART), and clinical characteristics of the HIV-1 infected partner (e.g., CD4 count, WHO stage).

Approved Operational Copy

Facility level factors would be assessed. This would include the impact of training on quality of service delivery and perceived changes by clients at the service sites, effective communication strategies, adherence counselling, use of centralized vs decentralized services for drug access, time spent and costs for travels to facility. The study shall also assess the impact of use of home self-test kits (OralQuick®) for partner recruitment and uptake of PrEP and TasP by serodiscordant couples. See the attached Protocol B.

Community level factors to be measured include the role of public communication and the impact of the designed community engagement programme on stigma and perception about the use of PrEP by community members. A specific detailed protocol will be developed to outline the community engagement methodology and the plan to assess the impact of the programme on PrEP uptake and adherence.

The primary outcome of this objective will be data and assessments about the factors that support or create barriers to clients' use of PrEP and partner's use of TasP.

Objective 3.2.2: Assess user preferences for ART initiation and PrEP among high-risk HIV-1 serodiscordant couples

Understanding couples' preferences for and concerns about antiretroviral-based HIV-1 prevention is of utmost importance at this time when guidelines for its use are still being formulated.

Interviewer-administered questionnaires will be used to collect data at baseline and quarterly. The questionnaire will specifically ask about a couple's willingness to use PrEP or ART for prevention and which method is preferred. Data regarding couples' reasons for their choice and their concerns about both methods will be collected both through the questionnaire and through mixed methods.

The primary outcome of this objective will be data and assessments about serodiscordant couples attitudes towards ART and PrEP as for preventing HIV-1 transmission.

Objective 3.2.3: Measure the adherence to PrEP among HIV-1 uninfected partners in serodiscordant couples.

Understanding adherence to PrEP is a critical research priority, particularly when PrEP is delivered outside of a controlled clinical trial setting and is being used as a bridge to ART for the infected partner. Brief adherence counseling for all participants will be delivered at baseline and then quarterly thereafter. The content of the adherence counseling will be derived from developed counseling messages and procedures defined for this study protocol. The messages will be refined as the project evolves. The role of individual risk aversion and adherence would also be measured (see full details of the study in Protocol C attached)

The primary outcome of this objective will be data on PrEP adherence. This will be in the form of clinic-based pill counts at follow-up visits and MEMS cap data on how frequently the pill bottle was opened.

Objective 3.2.4: Look for evidence of risk compensation by HIV-1 serodiscordant couples on PrEP or TasP.

Risk compensation when using PrEP occurs when patients, perceiving PrEP to reduce their risk of HIV acquisition, start to engage in riskier behaviors or to reduce their use

of other protection methods such as condoms. Clinical trials on PrEP have not found significant evidence of risk compensation, but the possibility cannot be ruled out in real-world practice.

The primary measure of this objective is reduction in rate of use of condom per couple on PrEP; and rate of increase in STI diagnosed per couple on PrEP. This data will be generated from the clinical data and the behaviour surveillance data.

Objective 3.2.5: Measure the probability of participants developing a resistance to Truvada during the course of the study.
PrEP and TasP studies have tried to address the issue of drug resistance to tenofovir and emtricitabine. Studies in HIV-1-infected individuals followed small numbers of patients for a short time. Very few cases of resistance have been reported, but participants in these clinical trials are monitored under rigorous clinical trial conditions that do not replicate real world events. Studies in macaques were well designed, but used SHIV, which has an attenuated course of infection.[56] The available information suggests that the probability of emergence of drug resistance is small. Infections that occurred despite use of PrEP had reduced peak viremia, which could reduce HIV transmissibility. Mathematical modeling suggests that, although transmitted drug resistance may under some circumstances increase, the benefits of PrEP outweigh the risks associated with resistance. Tenofovir and emtricitabine are, however, recommended in first-line treatment drugs in Nigeria. It is therefore important to confirm the expected low incidence of drug resistance in daily practice. This demonstration project will conduct a surveillance of drug resistance to Truvada in patients who take PrEP.

The primary measure of this objective is an estimate of the prevalence of ART resistance that developed in patients who seroconverted while using PrEP.

Objective 3.2.6: Estimate the resources that would be needed to provide PrEP and TasP for other high-risk groups.
It is expected that access to the prevention program will be opened to other high risk groups as the program is being rolled out. The cost-effectiveness of providing PrEP and TasP in other high risk groups, such as female sex workers and men who have sex with men, needs to be estimated as decisions are being made about scaling up the project.

The primary measures of this objective is an estimate of cost per life year gained and cost per infection averted for female sex workers and men who have sex with men.

4.0 Population

A sample with a size of 600 heterosexual HIV-1 serodiscordant couples will be recruited from ART sites with family planning clinics in three States in Nigeria. Couples who had not

56. van de Vijver DA, Boucher CA. The risk of HIV drug resistance following implementation of pre-exposure prophylaxis. Curr Opin Infect Dis. 201;23(6):621-627

.

had prior access to PrEP and couples whom the HIV positive index partner has not been sero-suppressed despite having commenced the use of ARV will be recruited for the study.

5.0 Study Sites

The ART sites currently supported by FHI360/SIDHAS and the Harvard/PEPFAR projects will be considered. These sites are the Federal Medical Centre at Nnewi in Anambra State, the Federal Medical Centre in Calabar, Cross Rivers State and the University of Jos, Plateau State. The use of ART sites for study conduct is important so as to ensure ART access by HIV positive partners who are eligible for ART.

One approach to programmatic scale-up of PrEP access within the public sector health service in Nigeria is to integrate its provision into existing service delivery. Integrating HIV prevention into routine outpatient services has several advantages, including:

- Clients would be able to access care and services without undue stigma that is associated with attendance of special clinics like the HIV clinic. Outpatient services are widely available in Nigeria
- The model would enable the project learn about how to provide HIV services as an integrated health care service which can facilitate unbiased attention to both male and females who attend the hospital for health care.

Integrating HIV prevention into HIV care services also has several advantages, including:

- Trained specialized care providers who understand HIV care and service provision would learn to incorporate PrEP and TasP into existing HIV prevention, treatment and care services.
- The HIV index partner can be better managed and monitored over the long term.

Empirical evidence is needed to assess whether integrating PrEP provision into outpatient care services and HIV treatment services can achieve similar levels of safety and PrEP use as observed in the various clinical trials. The study design however, would enable real time changes to be made to implementation plans based on outcomes of the formative assessments as more effective strategies for PrEP access by study populations are identified.

6.0 Study Services

Participants will receive baseline and quarterly individual and couples HIV-1 counseling, condoms, risk reduction counseling, and syndromic management of sexually transmitted infections according to local guidelines. HIV-1 infected partners will have quarterly monitoring of HIV-1 clinical status and 6-monthly CD4 counts and will be referred for HIV-1 care based on national HIV-1 care guidelines. Counseling on the importance of not sharing study medications within the partnership will occur at quarterly visits. To reduce interference with the real-world behaviors of the participants, tracing will occur only for follow-up of safety issues and for HIV-1 assessment at the end of the study, but not for

Approved Operational Copy

completion of routine visits and PrEP refills. This should allow a more realistic assessment of uptake and adherence.

7.0 Eligibility

7.1 For heterosexual couples

- Sexually active (defined as having had vaginal intercourse at least 6 times in the previous three months)
- Willing to enter the study as a couple and intending to remain as a couple for the next 12 months
- Has not been on PrEP medication prior to enrollment in study

7.2 For HIV-1 uninfected members of the couple (partner participants)

- Able and willing to provide written informed consent
- HIV-1 uninfected based on negative HIV-1 rapid tests, both at study screening and at the enrollment visit
- Adequate renal function, defined by normal creatinine levels and estimated creatinine clearance ≥60 mL/min
- Not currently pregnant or breastfeeding
- Not currently enrolled in an HIV-1 prevention clinical trial
- Not currently using PrEP
- Enrollment of individuals with active and serious infections or active clinically significant medical problems will be at the discretion of the site investigator

7.3 For HIV-1 infected members of the couple (index participants)

- Able and willing to provide written informed consent
- HIV-1 infected based on positive rapid HIV-1 tests, according to national algorithm
- No history of WHO stage III or IV conditions
- Not currently using ART
- Currently using ART but viral load still higher than 5000 copies/ml
- Not currently enrolled in an HIV-1 treatment study.
- Current pregnancy or breastfeeding do not disqualify a candidate who is infected.

8.0 Sample Size

A total of 600 couples will be enrolled from the three study sites. Enrollment will be competitive. All couples will participate in the longitudinal cohort study. A subset of up to 60 couples per site will be invited to participate in qualitative in-depth interviews and focus group discussions.

Approved Operational Copy

9.0 PrEP Medication

Tenofovir disoproxil fumarate (or TDF, 9-[(R)-2-[[bis[[(isopropoxycarbonyl)oxy]methoxy]phosphinyl] methoxy]propyl]adenine fumarate) and emtricitabine (or FTC, 5-fluoro-1-(2R,5S)-[2-(hydroxymethyl)-1,3-oxatholan-5-yl]cytosine) are reverse transcriptase inhibitors that have been approved for the treatment of HIV-1 infection in humans in Nigeria. A fixed-dose, oral co-formulation of FTC with TDF, which carried the trade-name Truvada®, will be used in this study. PrEP will be prescribed for once-daily use. Study medication will be donated by Gilead Sciences.

10.0 Recruitment

Each site will develop local recruitment and screening methods that operationalize protocol-specified requirements for eligibility determination in a manner that is tailored to and most efficient for the local study setting and target study population. The design of local recruitment and screening methods would be informed by the outcome of the PrEP formative study[57].

Recruitment strategies will include partnering with existing HIV counseling and testing (HCT) centers and CSOs working with families and couples, public promotion of couples HCT by well-known figures and community organizations such as churches and mosques, and community mobilization around couples HCT promotion (during Valentine's Day and World AIDs day).

Recruitment materials will educate couples about the probability of being HIV-1 serodiscordant based on available data, and risks of unknown HIV-1 serodiscordancy in terms of transmission to the HIV-1 uninfected partner, and will emphasize the benefits of couples HCT with specialized counseling services. Couples may be recruited for possible inclusion in the study through referrals from HCT centers and other community-based organizations, direct outreach, or other activities conducted at the study sites.

Regardless of the recruitment source, each partner in each couple will provide independent informed consent for screening. The screening process will proceed in a step-wise manner for both partners until either all screening procedures are completed or one or both of the partners is determined to be ineligible.

Although all required screening procedures may be completed in as few as two visits for each partner, additional visits may be conducted as needed (for example, if one or both partners want more time to consider whether to enroll in the study). At least one screening visit and the enrollment visit must be attended by both partners of a couple together, and at least one couple counseling session must take place during the screening process. There is no time limit on the screening process.

For those couples found to be eligible for the study, informed consent for study participation and enrollment in the study may proceed on the same day when eligibility is

57 There is an ongoing PrEP formative study that would enable stakeholders engage with the design of appropriate communication and recruitment strategies for male and female HIV serodiscordant couples for this study.

Approved Operational Copy

determined. Each partner will be asked to provide independent informed consent for study participation.

11.0 Study Procedures

It adopts the Partner's PrEP study approach as discussed at the WHO Demo Meeting in July 2012.[58] The anticipated total study duration is approximately 24 months, with accrual requiring approximately 6 months and follow-up continuing for approximately 18 months after the end of the accrual period. Potential study participants will be screened for eligibility and eligible participants will be enrolled in the study within 30 days of screening. Visits will take place at screening and enrollment, a visit 1 month after enrollment, and then quarterly thereafter, for up to 18 months.

11.1 Community education and mobilization

In line with outcomes of the PrEP formative research, and lessons learnt from past HIV prevention trial efforts, community engagement is key and critical. This study will build on the lessons learnt from the NICCAV project, a HIV vaccine demonstration project in Nigeria. It will adopt its community engagement structure for this study. The project will work with a not-for-profit organization as an operations partner for the community mobilization program for the project. The organization will build the capacity of other identified community based partner organizations working in the various communities hosting the trial sites to conduct public education and support for the project. The capacity building effort will be such that seeks to have the partners integrate public education on PrEP and biomedical HIV prevention as an integral component of the organizational activities. This would ensure sustained public education effort long after the conclusion of the demonstration project.

11.1.1 Project Community Advisory Board (CAB)

Lessons learnt from the NICCAV project and other projects of such magnitude in Nigeria will be used to cultivate community support and assist in the establishment of an active Community Advisory Board (CAB) with representative from various community sectors. Consultations will be held with key stakeholders, and communities at the site of the project will continually be educated about HIV, AIDS, biomedical HIV prevention, PrEP and the role of research in HIV prevention. Once established, the CAB will play an advisory role to the project. The capacity of the members of the CAB will be built by the reference organization engaged with the NICCAV project in Nigeria so that they are involved in discussing the research from an informed perspective. A structured capacity building process that will enable members give constructive feedback on research protocols, monitor the project as community representatives, and disseminate information about the research to their respective constituencies will be developed. Lessons learnt and best practices from other studies will be shared with communities at the three study sites to ensure full community participation and retention.

Operational Copy

58 On the 21st and 22nd of July, 2012, WHO hosted a meeting of the PrEP working group to develop consensus on a framework for country level protocol development for Pre-Exposure Prophylaxis (PrEP) demonstration projects. The working group reached consensus on elements to be included in country demonstration project protocols. The meeting recommended that demonstration projects should follow the Partners PrEP demonstration project model

11.1.2 Community education

The Project Community Liaison Officer will work with the CAB and local community groups to disseminate culturally and linguistically appropriate information pertinent to the study within the communities at the study sites. Educational materials will be developed through a community consultative process to ensure culture representation and language appropriateness. The community engagement efforts would address community norms that would otherwise impede patient use of and adherence to antiretroviral-based HIV-1 prevention services.

11.1.3 Interaction with the media

The communication working group, hosted by the Corporate Communication Unit of NACA, would work with the media to promote public awareness and education about the PrEP study and public support for PrEP use by sero-discordant couples. Public communication mechanism shall be designed based on specific community needs. The electronic and print media shall be engaged extensively for this project. In addition, the local town criers in target study communities would be engaged to facilitate local community announcement of the study.

11.2 Study site preparation

As part of the study intervention, a QI approach will be used to assist the service delivery outlets expand their current services to include PrEP and TasP provision. An experienced QI advisor will work with the staff at the service delivery outlets to conduct a gap analysis of existing service provision prior to the enrollment of study participants at these sites. External and internal ideas will be carefully vetted to improve the quality of service delivery in the areas of family planning counseling and contraception provision, STI and HIV counseling and treatment, and general clinic processes (e.g. clinic flow, documentation, follow-up). The QI advisor will undertake ongoing monitoring of the quality of each of these services. Once the initial QI process has been completed at the participating service delivery outlets, a site initiation assessment will be undertaken to ensure procedures are in place for the study including procedures for dispensing PrEP and TasP. Once each service outlet meets the requirements for site initiation, study participants will be enrolled and the same QI approach will be used to integrate reliable PrEP and TasP provision and monitoring into the service delivery programs.

Specific steps used in the QI approach to be implemented in the hospitals that hosts the service delivery outlets include:

- Acknowledgement that service delivery outlet (out-patient clinic or HIV service delivery clinic) need to be strengthened and improved
- Clear commitment by facility-based staff to specific time-limited aims to ensure improvements to the quality of services to be provided
- Completion of a facility audit to identify nature of challenges and bottlenecks in system that impede service delivery
- Establishment of a clinic-based multi-disciplinary “improvement team”
- Goals and timelines for rapid improvement of outcomes set

Approved Operational Copy

- Mapping of relevant clinic-based processes for service provision with identification of critical milestones and timelines
- Development of systems tools to support the QI process
- Development and testing of specific changes to the system using the Model for Improvement and PDSA cycles
- Establishment of logs and data collection forms to monitor progress
- Rapid feedback of clinic data to enable the clinic QI team to identify ongoing challenges and develop solutions
- The introduction of PrEP and TasP into services will build on QI strengthened service delivery.
- Staff will be provided with detailed information on what is known about PrEP and TasP and strategies for providing individualized user support.

With support from the experienced QI advisor, the clinic QI team will:

- Set clear goals for service delivery improvement
- Map out the critical steps required for provision of PrEP and TasP to clients
- Develop system tools to support PrEP and TasP provision including data collection tools to monitor progress
- Oversee implementation of PrEP and TasP delivery plan
- Review data and feedback to family planning staff to develop solutions to challenges

This two-step approach of initially strengthening the services and introducing PrEP and TasP using a QI framework will create a cadre of service providers who can remain vigilant about the quality of services provided and cope with unexpected or unanticipated situations, in contrast to a more traditional prescriptive, top-down approach of service delivery.

11.3 Supply, storage and dispensing of PrEP drugs

Throughout the study duration, PrEP medication will be supplied free to participants on the project. It is anticipated that Gilead will supply all the medications for the study free. All study drugs will be produced under Good Manufacturing Practices (GMP) conditions. The study drugs will be acquired from Gilead thorugh the procurement team of NACA and distributed to all the study sites.. The Study Pharmacist will maintain full accountability records in accordance with Good Clinical Practice (GCP) and legal requirements. These will include pharmacy specific participant logs as well as stock accountability records.

Study product will be securely stored at controlled room temperature below 30°C until required for administration. PrEP should be stored away from direct sunlight.

PrEP will be dispensed directly by designated individuals to enrolled study participants as per Standard Operating Procedures (SOPs) in quantities expected to be sufficient until the participant's next follow-up visit. If a participant needs additional supplies between visits, (s)he will be instructed to contact the study clinic to request additional supplies. Study

Approved Operational Copy

participants will be asked to return to the clinic with tablet bottles at each visit. All returned drugs will be reconciled with the number dispensed to the participant and the outcome logged. Further details of the dispensing procedures will be outlined in the Study Specific Pharmacy Procedures Manual. In addition, the recordings from the MEMS Cap will be analyzed and used to corroborate the self-reported adherence and pill counts.

11.4 Recruitment, screening and enrollment

Eligible participants will be enrolled over approximately 12 months. At regular intervals, the Principal Investigators, in consultation with the study team, will assess progress in accrual and retention at each of the sites and may reallocate enrollment numbers and targets across the sites, as deemed necessary to achieve the goals of this trial efficiently. All potential participants will be screened, enrolled and escorted to the respective family planning services, for completion of enrollment visit procedures.

At screening, demographic and behavioral information will be collected, along with laboratory results to establish the risk score and participant eligibility (for HIV-1 uninfected partners – serum creatinine, hepatitis B surface antigen; for HIV-1 infected partners – CD4 count, plasma HIV-1 viral load; for both partners – HIV-1 rapid testing according to national algorithms).

11.5 Monitoring adherence and safety

At enrollment, HIV-1 testing will be performed for HIV-1 uninfected partners, to confirm eligibility (HIV-1 seronegative at the time of study start). Couples will be counseled about ART and PrEP. HIV-1 uninfected partners will be offered PrEP; HIV-1 infected partners will be counseled about ART guidelines and will be offered TasP, or referred/initiated on ART if they are eligible at the time of enrollment. HIV-1 infected partners will continued to be counseled on the use of TasP as long as (s)he is eligible to use TasP during the study period. HIV-1 uninfected partners who have symptoms potentially consistent with acute HIV-1 infection (fever, rash, pharyngitis) will have enrollment deferred for 2 weeks at which time repeat serologic testing will be performed (and, if positive, will result in study exclusion).

Adherence, risk reduction and contraceptive counseling will be completed. Adherence of HIV-1 uninfected participants will be measured by self-report and pill counts. In addition, the MEMS cap would be used to complement data generated in the clinic on self-reported pill use. The MEMS cap would generate information on the daily use of pill through electronically capturing data on the frequency of opening the drug bottles. Outcomes of the adherence profile generated from self-reported pill counts and the MEMS cap would be used to counsel patients on how to improve or adhere to drug-use adherence patterns.

Additionally, behavioral and medical history data as well as data on participant preferences regarding antiretroviral-based prevention strategies and fertility intentions will be collected during one-on-one interviews with staff counsellors. Physical examinations for both members of the couple will be conducted. For the woman, physical examination will include pelvic examinations, including naked eye examination of the external genitalia and speculum examination of the vagina and cervix. For males, physical examination will be limited to naked eye examination of the external genitalia.

Approved Operational Copy

Syndromic diagnosis and management of STIs will be done. Counseling and clinical care for STIs at the study sites will be in accordance with the Federal Ministry of Health's guidelines. Participants with STIs will be encouraged to refer their partners for treatment.

At enrollment HIV-1 uninfected women will have a pregnancy test to confirm eligibility (must not be pregnant to complete enrollment). Women will be asked about pregnancy at every scheduled follow-up visit and pregnancy testing will be done when clinically indicated (e.g., missed menses, participant request); PrEP will be continued during pregnancy and breastfeeding. In addition, pregnant women will be advised to continue with their follow up visits. When these participants no longer have a positive pregnancy test, the pregnancy outcome will be documented.

Every 6 months, in addition to regular quarterly visit procedures, CD4 counts and plasma viral loads will be done for HIV-1 infected partners.

Clinical safety will be assessed pre-, during- and post-enrollment. Laboratory safety assessments will be done at enrollment, month 12, study exit and additionally if indicated. At these visits, blood samples will be collected for urea, electrolytes, creatinine, liver function tests, full blood count, calcium and phosphate levels.

All HIV-1 infected partners who are interested in starting ART independent of their becoming eligible for ART under the current national guidelines, will be offered ART. Once the HIV-1 infected partner has been using ART for 6 months, PrEP will be discontinued for the HIV-1 uninfected partner, after counseling to the couple.

During follow-up, HIV-1 infected and uninfected participants will complete a quantitative interview to capture evolution in preferences to uptake of antiretroviral-based HIV-1 prevention and fertility intentions and any risk compensation behaviours. Questionnaires will be administered separately to both members of couples.

For participants who are initially uninfected but who seroconvert during the study, PrEP will be discontinued. Their blood specimen will be taken and drug resistance profiling will be conducted. This is to assess if any drug resistance has developed to Truvada and what drug regimen the study participant is sensitive to. Seroconverters will have a visit within 1-month after they seroconvert and then follow-up in the study will be completed for both members of the couple. These visits will include risk reduction and contraceptive counseling, collection of behavioral and medical history data during one-on-one interviews, and collection of blood specimens for assessment of CD4 count, HIV-1 plasma viral load levels from both partners and HIV resistance profiling. Those who seroconvert will be enlisted to have access to ART whenever (s)he is ready to start drug treatment.

Interim visits may occur at any time during the study. All interim contacts and visits will be documented in participants' study records and on applicable CRFs. If a study participant presents to an interim visit with symptoms suggestive of acute HIV-1 infection syndrome, rapid HIV-1 testing will be performed, as at a scheduled visit, and study medication will be withheld if a positive rapid test is documented.

HIV-1 testing for index (HIV-1 infected) participants at screening and for partner (HIV-1 uninfected) participants at screening, enrollment, and follow-up – will be performed in line with national HIV-1 testing algorithms for Nigeria. HIV-1 seroconversions will be

Approved Operational Copy

confirmed using HIV-1 EIA. HIV-1 testing will be accompanied by couples and/or individual counseling, depending on attendance of the index (HIV-1 infected) participant at a study visit. All counseling and testing approaches will be in accordance with national HIV-1 counseling and testing guidelines.

Participants infected with hepatitis B virus (HBV) at enrollment will be closely monitored clinically and using laboratory diagnostics especially during episodes of product hold. Any participant needing further treatment for HBV will be referred to a health care provider for further follow-up.

All data collection will be conducted in a private room in the participant's preferred language, according to the participant's fluency and preference.

11.6 Co-enrollment guidelines

Participants in this study may not take part in any other concurrent research studies that would interfere with the objectives of this study. The determination of whether participation in another study would be exclusionary for a given participant will be made by the Principal Investigators. Approved co-enrollment in other concurrent protocols will be documented.

11.7 Participant retention

The Protocol Team will track retention rates. Once a participant is enrolled in the study, study staff will make every reasonable effort to ensure adequate locator information is available for follow-up tracking. Participants will be reminded of their clinic visits using sms messages sent on their telephones. A missed visit will prompt a single telephonic reminder, where the participant is spoken to directly and reminded of the clinic visit. A maximum of 3 reminders will be made. Retention efforts will be standardized across study sites.

11.8 Participant withdrawal

Participants may voluntarily withdraw from the study for any reason at any time. Designated study staff may also withdraw participants from the study in order to protect their safety. Participants may also be withdrawn if the National Ethics Committee terminates the study prior to its planned end date. Every reasonable effort will be made to complete a final evaluation of participants who withdraw or are withdrawn from the study. Study staff will record the reason(s) for all withdrawals in participants' study records.

11.9 Adherence counseling and case management

Adherence counseling will be provided to study participants upon enrollment and additionally at each study visit. Adherence counselors / nurses will be provided with a set of job aids, including a reference guide to assist with and standardize adherence support counseling. Techniques based on motivational interviewing will be used to address such topics as participant-centered strategies to remember to use the products daily, to ensure the availability of the products both in the home and away from home, and to identify and discuss various challenges and situations that may impede product use. Counseling will include reminders to contact study staff with questions about PrEP use and requests for

Approved Operational Copy

additional supplies. For participants experiencing adherence challenges, every effort will be made to identify customized strategies to increase their rates of product use throughout the course of the study. Data on adherence will be collected at each study visit via brief interviewer administered instruments.

11.10 Support for adherence

Partners in a couple will serve as the PrEP and TasP adherence supports for each other. Participants will be reminded of the need to take their drugs through the use of sms messages sent to their telephones. The Nigerian Communications Commission (NCC) reports a mobile phone subscription rate of 94.7% with a national teledensity of 72.3 as of May [59] (Teledensity is the number of telephones per 100 people.) Notably, there has been a sustained upward trajectory of mobile phone subscribers, and the teledensity is expected to exceed 100 within the next three years. This means that mobile phones are already very common; so this intervention will not require additional infrastructure. Computer based monitoring systems are user friendly, culturally appropriate, and convenient for the target population, as they can use their personal mobile phones.[60] We expect that this intervention will increase adherence to daily schedules for PrEP use, refill appointments and clinic schedules. It is expected to be an effective and cost-efficient intervention.

11.11 Support for HIV positive partners

Conventional approaches for supporting HIV positive clients in the study sites will be adopted for this study. This would include: ongoing HIV counseling and risk reduction counseling in line with the National guidelines; couples counseling, and counseling on family planning and child bearing. Clients will also be referred to support groups. These conventional approaches will be augmented with the use of sms reminders for drug adherence for all study participants who opt for use of TasP for HIV prevention.

11.12 Suspending or discontinuing PrEP

Study participants will be discontinued from using PrEP by the Principal Investigators and their designees in the event that they experience a Serious Adverse Event (SAE) that is judged by the study clinician or designee to be related to PrEP use (see Section 8.2). Pregnancy will not be an indication for the discontinuation of the drug because risk of HIV transmission is high in pregnancy, and the study drugs are not contraindicated in pregnancy.

For participants who temporarily discontinue PrEP use, every effort will be made to complete all procedures specified in the protocol (except study product dispensing procedures). Designated staff will document all changes in PrEP regimen, and the reason for the change, on applicable Case Report Forms (CRFs).

Approved Operational Copy

59 Nigerian Communications Commission. 2012. Subscriber Data. http://www.ncc.gov.ng/industry-statistics/subscriber-data.html. Accessed, July 2012.

60 Curioso WH, et al. Design and Implementation of Cell PREVEN: A Real-Time Surveillance System for Adverse Events Using Cell Phones in Peru. AMIA Annu Symp Proc 2005; 176-180

11.13 Concomitant medications

Enrolled study participants may continue use of all concomitant medications, including prescription, nonprescription, traditional, and other preparations during this study. All concomitant medications used by participants during the study will be reported on applicable CRFs.

12.0 Safety

12.1 Adverse events and reporting requirements

An AE is defined as any untoward medical or social occurrence in a clinical research participant whether or not it has a causal relationship with the study product. (Study product refers to Truvada pills.) Information regarding symptoms or conditions that occur during the screening period will be recorded in the participant's medical history as pre-existing conditions. All new or worsening symptoms or conditions that occur following randomization will be considered AEs and will be recorded on the appropriate CRF.

12.2 Adverse event reporting

Study participants will be provided contact telephone numbers and instructed to contact study staff to report any AEs they may experience, except for life-threatening events for which they will be instructed to seek immediate emergency care. Depending on the severity of the event, the study staff will instruct the participant to present to their assigned study site (for mild events) or to a hospital casualty department (for serious events) for immediate evaluation. With appropriate permission of the participant, records from all non-study medical providers related to AEs will be obtained and required data elements will be recorded on study CRFs. All participants reporting an AE will be followed clinically, until the AE resolves (returns to baseline) or stabilizes. AEs that are ongoing at the time of study exit will be followed up for up to 30 days after study exit and then, if not resolved, will be referred to a health care provider for further follow-up. Designated study staff will determine the severity of the AE and document it on the appropriate CRF. Each adverse event that the participant is aware of will be graded for severity using the most current DAIDS severity grading system.

For each AE, an assessment of the relatedness to the study product will be made using the criteria and scale as outlined in the SSP manual. All AEs will be captured regardless of the association or otherwise to PrEP and reported on the appropriate CRF in accordance with the SSP. All AE reports will contain at least the date the AE occurred, a brief description of the event, the relationship to study product, the action taken, the outcome, date resolved, and the seriousness of the event.

12.3 Serious adverse event (SAE) reporting

An SAE includes any experience that is fatal or life-threatening, results in persistent or significant disability/incapacity, requires or prolongs hospitalization, or is a congenital anomaly. A life-threatening AE means that the participant was, in the view of the designated study staff, at immediate risk of death from the condition as it occurred. Notification of deaths will be recorded by reflecting the medical condition that led to the

Approved Operational Copy

death on the appropriate CRF. Reporting SAEs may require additional detailed reports and follow-up. All serious adverse events will be reported to Gilead, the National Agency for the Food and Drug Administration and Control (NAFDAC) and the National Health Research Ethics Committee (NHREC).

12.4 Safety monitoring

Designated study staff will be responsible for continuous close safety monitoring of all study participants. The study statisticians will prepare routine study progress reports for review by the Protocol Team. In addition, the study statisticians will prepare routine study progress reports which include reports of AEs experienced by study participants.

13.0 Data management and analysis

13.1 Data analysis

A Statistical Analysis Plan (SAP) will be developed. This will outline the planned analyses. Any deviations to be made from the plan will be documented in the SAP.

For the primary objective 3.1.1, the outcome variable to be measured will be the uptake of antiretroviral-based HIV-1 prevention program. Data collected will include all sero-discordant couples recruited into the study and will be followed up longitudinally. The number of new infections averted through the use of PrEP and TasP by sero-discordant couples who accessed the ARV preventions compared with those who had access to programs but did not use the tools will be documented. The impact of various variables such as age, viral load of partner, region, coital act, use of condom on uptake of PrEP and TasP and on HIV sero-conversion will also be assessed.

13.2 Data management

Data will be collected on standardized CRFs which will be developed by the study team. Site study staff will be trained in the correct completion of CRFs. If data entered on the CRFs are taken from an external source (e.g., laboratory reports, patient records), the source documents will be maintained in the participant's medical chart or study file at the site, and will be available for review. The CRFs will be stored electronically in the study database. All data entry will be done manually.

Questions arising during validation of the data will be recorded in quality control (QC) reports, which will be sent to the sites on a regular basis. Any questions that result in a change to the database will be documented and attached to the original CRF. The data management center staff will perform periodic quality control and validation checks on the data. Database files will be password-protected and access to the files will be limited to authorized study staff members only. All data will be backed up at regular intervals, and backups will be stored in secure areas with limited access.

The original CRFs and related documents will be stored securely at the sites, both during and after the completion of the study. At all sites the forms will be stored in locked cupboards in a secure room with restricted access. Upon completion of the study, the close-out site monitoring visit and finalization of the database for analysis, the original forms will

Approved Operational Copy

placed in long term storage. More detail on how the data will be managed will be contained in the study's data management plan.

14.0 Ethical Considerations

14.1 Regulatory and ethical review

This study will be conducted under the regulatory oversight of NAFDAC. It will be performed in accordance with International Conference on Harmonization (ICH) guidance and GCP standards. The study also will be conducted under the ethical oversight of NHREC. The study will only be initiated after it has been approved by the NAFDAC and NHREC. The study will be conducted in accordance with all conditions of approval by the ethics and regulatory committees.

14.2 Informed consent

Informed consent will be obtained from each study participant prior to screening and enrollment, in accordance with GCP guidelines. Participants will be provided with copies of their informed consent forms if they are willing to receive them. An impartial witness will be available for the entire informed consent process with any participant who is illiterate or whose literacy is limited. Documentation of the presence of a witness will be achieved through their signature on the informed consent document. Illiterate participants will indicate their consent via use of their mark (finger/thumb print) on the informed consent documents.

14.3 Risks

Risks associated with study participation are medical, social and confidentiality risks. Study participants may experience discomfort when having pelvic examinations and/or undergoing phlebotomy for this study. During phlebotomy, participants may feel dizzy or faint, and/or develop a bruise, swelling, or infection where the needle is inserted. Participants may become embarrassed, worried, or anxious when completing their HIV-related interviews and/or receiving HIV/STI counseling. They also may become worried or anxious while waiting for their HIV test results or after receiving HIV-positive test results. Trained study staff will be available to help participants deal with these feelings. Study personnel will make every effort to protect participant privacy and confidentiality, but it is possible that participants may disclose their HIV status to non-study participants and could be treated unfairly or discriminated against, or could have problems being accepted by their families and/or communities. Participants also could have problems in their partner relationships associated with use or attempted use of condoms and/or the study product.

Data on participant risk behaviors and the occurrence of other potential social harms will be collected from all participants. The Protocol Team will monitor trends in risk behaviors over time based on these data, as well as the occurrence of social harms, and initiate any required follow-up action.

Available clinical trial evidence indicates that Truvada is safe and well tolerated. There is a possibility of Truvada resistance in HIV strains from those who acquire HIV infection while using the study product. Limited resistance data showed that for eight participants in

Approved Operational Copy

Partners PrEP and three participants in TDF2 who had primary HIV infection when they joined the study, three cases developed HIV that was resistant to their PrEP drugs. One participant who took tenofovir alone in the Partners PrEP trial developed high-level resistance to tenofovir and one taking Truvada developed resistance to FTC, while one participant in the TDF2 trial developed resistance to both drugs. No participants who became infected with HIV after enrollment developed drug resistance.[61]

Some of the possible side effects of the study drug identified in both the Partners PrEP and TDF2 studies are increased levels of gastrointestinal symptoms (diarrhea, vomiting and especially nausea, which was over twice as common) when compared to participants taking placebo. There were no indications of kidney problems related to the drugs. TDF2, however, also measured bone mineral density (BMD), and observed a significant and continuing loss of BMD over the two years of the study, in contrast to participants taking placebo, who gained BMD.[62]

14.4 Benefits

14.4.1 To the individual

Study participants will benefit from access to PrEP, a product with demonstrated protection against HIV infection. Participants in this study will benefit from HIV education and prevention messages. They will receive free HIV, STI, and hepatitis serologic testing and free medical treatment for diagnosed curable STIs. Contraceptive method of choice will be available to all study participants. For participants who have a diagnosed curable STI, they will be offered free STI testing and treatment. For other medical conditions identified as part of the study screening and/or follow-up procedures, participants will be referred to other sources of care available in their community. Study participants will also receive condoms and risk reduction counseling. Participants who acquire HIV infection during the study will be referred to treatment centres for ongoing care and support including laboratory testing and monitoring of immune function, virological changes, tenofovir resistance, and access to antiretroviral therapy (ART) in accordance with national policies regarding HIV treatment.

The FGDs may also be an opportunity for those participants to voice out concerns regarding healthcare in their own communities, and potentially contribute to major, positive changes that would result from such conversation. This could give participants a feeling/sense of satisfaction having contributed to greater public good.

The results of the TDF2 study suggests the possibility of study participants who take Truvada having less risk of urethral STI symptoms when compared to those using placebo.[63]

14.4.2 To the community and public at large

Participants and others may benefit in the future from information learned from this study. Specifically, information learned in this study could enhance the scale-up and

[61] http://www.aidsmap.com/High-levels-of-adherence-reached-in-two-PrEP-studies-but-resistance-cases-show-need-for-rigorous-testing-before-prescribing/page/2442372/. Accessed 18th November, 2012

[62] Ibid.

[63] Ibid.

implementation of PrEP when Truvada is licensed for use for HIV prevention in Nigeria. The data generated from this study can enhance Truvada licensure for HIV prevention use in Nigeria.

14.5 Access to HIV-related care

14.5.1 HIV counseling and testing

HIV counseling will be provided to all potential study participants who consent to undergo HIV screening to determine their eligibility for this study, and to all enrolled participants at each study visit. HIV test results will be provided with post-test counseling. Male and female condoms will be provided to participants throughout the duration of their participation in the trial.

14.5.2 Care for participants identified as HIV-infected

Potential study participants who volunteer to undergo HIV testing as part of the study screening process may discover that they are HIV positive. Study staff will provide all HIV test results with post-test counseling. Potential study participant who have been identified as HIV positive will be referred to local AIDS treatment services which provides medical and psychosocial AIDS care and support. Their care will be monitored as part of the requirements for this study.

HIV-uninfected study participants who become infected during follow-up will be referred to preferred local AIDS treatment services - government or non-governmental HIV/AIDS care services - for ongoing clinical management and care.

14.6 Community involvement and consultation

The project will establish a Community Advisory Board (CAB) through a consultative process. CABs will be established at the sites where this study will be conducted. The CAB membership will include local community leaders, traditional leaders, leadership of local HIV/AIDS organizations, local health service provider representatives and HIV positive local community members. The community and local community based organizations will be involved in the preparation of sites for this study through active mobilization of the Project Community Liaison Organization (NHVMAS). Specifically, NHVMAS will build on its experience through the NICCAV project: it will inform, educate, mobilize and address the capacity needs of the community to enhance community input into the research process. The local CABs will play an active role as an interface between the researchers and community members, serving as advocates for the community's best interests and ensuring that the researchers are aware of any concerns within the community about the research being conducted. The CABs also play an important role in reviewing study educational materials, consent forms and translations of documents, which will be shared with study participants. The QI team in the intervention clinics will provide additional and ongoing input into this study from a provider and another key stakeholder perspective.

14.7 Confidentiality

Every effort will be made to protect participant privacy and confidentiality to the extent permitted by law. All research staff and study site health care providers will receive training on confidentiality as part of the capacity building trainings. The issue of

Approved Operational Copy

confidentiality will be reiterated and reinforced throughout the study period. Study-related information will be stored securely at the study's clinical research sites. All participant information will be stored in lockable file cabinets in areas with access limited to study staff. Data collection, process, and administrative forms, laboratory specimens, and other reports will be identified by a coded number only, to maintain participant confidentiality. All records that contain names or other personal identifiers, such as locator forms and informed consent forms, will be stored separately from study records identified by code number. All databases will be secured with password-protected access systems. Forms, lists, logbooks, appointment books, and any other listings that link PID numbers to other identifying information will be stored in a separate, locked file in an area with limited access.

Participant study data, as identified by PID number only, will not be released without the participant's written permission, except as necessary for review and monitoring by:

- Authorized study representatives
- NAFDAC
- NHREC
- Study Monitors

14.8 Study Discontinuation

This study may be discontinued at any time by NAFDAC, NHREC, or the Protocol Team (e.g., in response to recommendations from external regulatory agencies like World Health Organization or the study sponsors).

15.0. Laboratory Considerations

The study laboratory plan will include the procedures for specimen management (e.g., chain of custody, handling, labeling and transport), assay procedures, proficiency testing and quality assurance procedures, and specimen storage procedures.

15.1 Laboratory specimens

The following types of specimens will be collected for testing:

- Urine for pregnancy testing
- Blood for hematology and chemistry
- Blood for HIV testing by rapid tests, confirmatory RNA PCR assays, Western blots and/or ELISAs
- Blood for HBV testing
- Blood from suspected seroconverters for Truvada resistance assays

All the above specimens will be collected following Good Clinical and Laboratory Practice standards and as described in the SOPs for collection of specimens.

Approved Operational Copy

15.2 On site testing

The study laboratory plan will detail the procedures to be followed for on-site testing as well as proficiency testing for all on-site testing.

15.3 Laboratory quality control and quality assurance procedures

The laboratories involved in the study will follow the quality assurance and quality control procedures outlined in the study laboratory plan. Periodic visits to the study clinics will be conducted to assess the implementation of on-site quality control procedures in a fashion that is consistent with real life practice. This will include maintenance of laboratory testing equipment, use of appropriate reagents, proficiency testing records and quality checks of on-site testing procedures.

16.0. Administrative Procedures

16.1 Protocol compliance

The study will be conducted in full compliance with the protocol. Amendments to the protocol will be required to follow an SOP which stipulates the levels of approval required prior to submission to regulatory bodies and the steps to be followed prior to implementation of a protocol amendment.

16.2 Protocol deviations and violations

Protocol deviations and violations are broadly defined as any departure from the procedures described in the study protocol. They may impact subject safety, affect the integrity of study data, affect subject's willingness to participate in the study, and/or provide evidence of willful or knowing misconduct or non-compliance on the part of the site investigator(s). Such protocol deviations will be documented and reported. Protocol deviations and violations may be identified by any of the study staff or by the study monitor. The procedures for documenting these will be specified in the monitoring plan.

Some examples of protocol violations include:

- Omission or inadequate administration of informed consent
- Inclusion/exclusion errors, including legal age limit
- Missing or incorrectly timed study procedures and assessments
- Failure to discontinue product use due to protocol criteria

In an emergency, the Investigator may make departures from the protocol to eliminate an apparent immediate hazard for a particular participant. In such a case, (s)he will notify NHREC in writing as soon as possible and document reasons for the violation (unless solely caused by participant noncompliance such as not attending for study visits).

16.3 Quality assurance

Quality assurance in the trial will be undertaken according to the Study Quality Assurance Plan. The Quality Assurance Plan will include ongoing monitoring of study progress and

Approved Operational Copy

safety by the Protocol Team, study monitoring in accordance with GCP guidelines. The Investigators will allow study monitors to inspect study facilities and documentation (e.g., informed consent forms, clinic and laboratory records, other source documents, CRFs), as well as observe the performance of study procedures. The Investigators will also allow inspection of all study-related documentation by authorized representatives of NAFDAC and NHREC. A site visit log will be maintained at the study site to document all visits.

16.4 Study monitoring

Study monitoring will be conducted by the Protocol Clinical Monitoring Unit along with an external study monitor. Pre-initiation site monitoring will be undertaken to establish study site readiness for study initiation. Thereafter ongoing monitoring will be undertaken after enrollment of the first participants and at regular intervals thereafter. A site visit log will be maintained at the study site to document all visits. Monitor findings will be documented per study monitoring SOPs. The Principal Investigators will be notified of the findings. If the monitor discovers issues related to safety, (s)he is to report their findings immediately to the Principal Investigators or designee.

16.5 Study records

Complete, accurate, and current study records will be maintained and stored in a secure manner throughout the study. All study records will be maintained for at least 5 years after the termination of the study.

16.6 Use of information and publications

Presentation and publication of the results of this study will be governed by study sponsor's publication policy.

Approved Operational Copy
