# Supplementary figures and images for: Effectiveness of three delivery models for promoting access to pre-exposure prophylaxis in HIV-1 serodiscordant couples in Nigeria
Source: PLoS One. 2022 May 5;17(5):e0268011. doi: 10.1371/journal.pone.0268011 (PMC9070899; doi:10.1371/journal.pone.0268011)

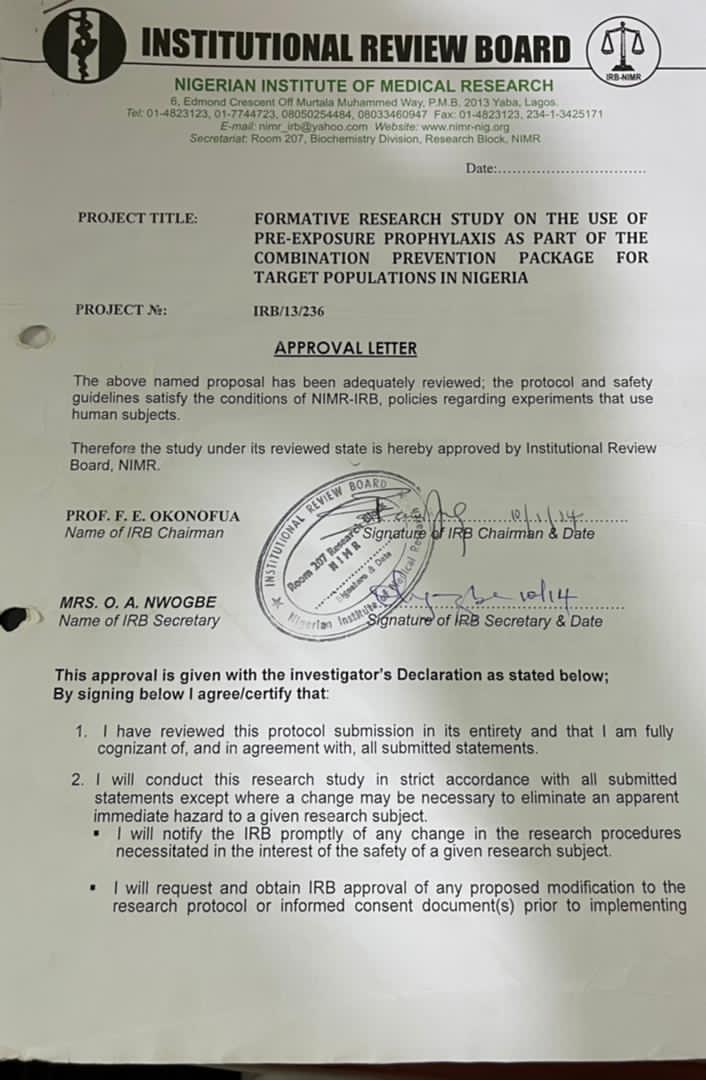

Supplement: S1 Fig — (JPG) [file pone.0268011.s004.jpg]

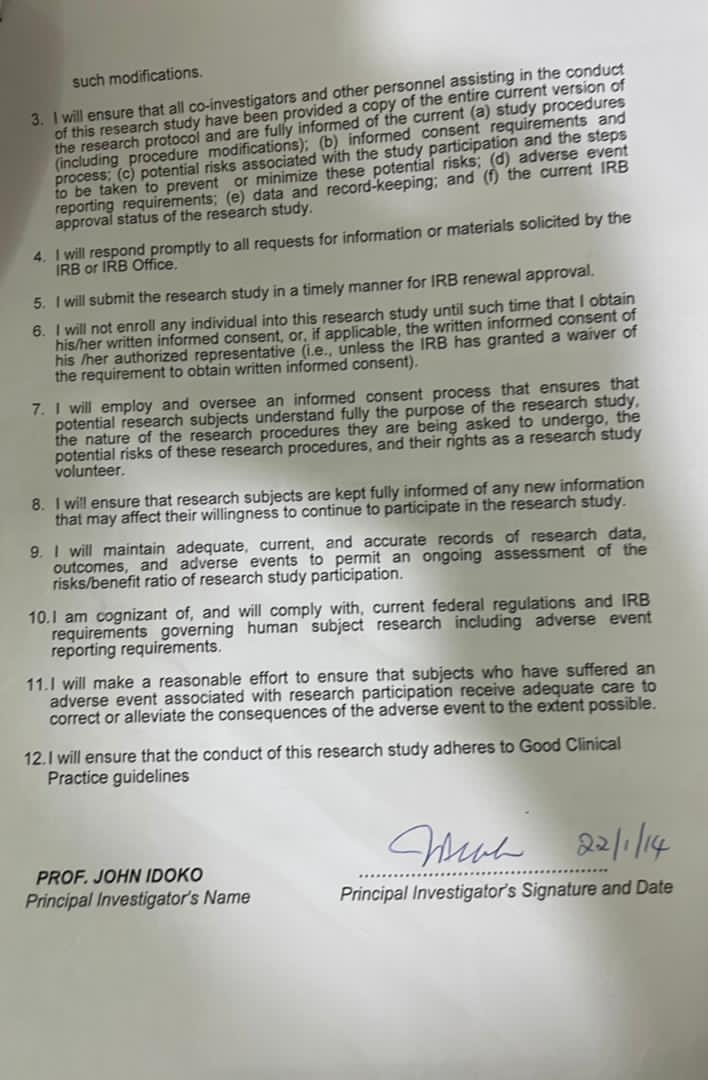

Supplement: S2 Fig — (JPG) [file pone.0268011.s005.jpg]
