# Supplementary material for: Effectiveness of three delivery models for promoting access to pre-exposure prophylaxis in HIV-1 serodiscordant couples in Nigeria
Source: PLoS One. 2022 May 5;17(5):e0268011. doi: 10.1371/journal.pone.0268011 (PMC9070899; doi:10.1371/journal.pone.0268011)
Supplement: Attachment — Submitted filename: Response to Reviewers.docx [file pone.0268011.s006.docx]

Letter of Response to Reviewers

Dear Reviewers,

Thank you very kindly for your highly professional review and constructive feedbacks. We have made revisions to the manuscript based on your comment. Also we have made clarifications where needed in the manuscript. Please see below in blue font colour responses to your comments.

Thank you.

Sincere regards,

John Idoko

Reviewers' comments:

Reviewer's Responses to Questions

**Comments to the Author**

1. Is the manuscript technically sound, and do the data support the conclusions?

The manuscript must describe a technically sound piece of scientific research with data that supports the conclusions. Experiments must have been conducted rigorously, with appropriate controls, replication, and sample sizes. The conclusions must be drawn appropriately based on the data presented.

Reviewer #2: Partly

Response to comment: The manuscript has been revised to better describe the study design.

2. Has the statistical analysis been performed appropriately and rigorously?

Reviewer #2: No

Response to comment: The statistical analysis has been revised and appropriate test statistics applied to the analysis.

3. Have the authors made all data underlying the findings in their manuscript fully available?

The [PLOS Data policy](http://www.plosone.org/static/policies.action#sharing) requires authors to make all data underlying the findings described in their manuscript fully available without restriction, with rare exception (please refer to the Data Availability Statement in the manuscript PDF file). The data should be provided as part of the manuscript or its supporting information, or deposited to a public repository. For example, in addition to summary statistics, the data points behind means, medians and variance measures should be available. If there are restrictions on publicly sharing data—e.g. participant privacy or use of data from a third party—those must be specified.

Reviewer #2: Yes

4. Is the manuscript presented in an intelligible fashion and written in standard English?

PLOS ONE does not copyedit accepted manuscripts, so the language in submitted articles must be clear, correct, and unambiguous. Any typographical or grammatical errors should be corrected at revision, so please note any specific errors here.

Reviewer #2: Yes

5. Review Comments to the Author

Please use the space provided to explain your answers to the questions above. You may also include additional comments for the author, including concerns about dual publication, research ethics, or publication ethics. (Please upload your review as an attachment if it exceeds 20,000 characters)

Reviewer #1: The objective of this study was to evaluate the effectiveness of three models for pre-exposure prophylaxis (PrEP) service delivery to HIV-1 serodiscordant couples in Nigeria.
Below are some comments:
- The experimental unit of this study was site as randomization was done at site level. However there was only a single site for each treatment arm. This essentially makes the study arm un-testable utilizing the randomization (i.e. no possible inference in a randomized setting based on sampling unit). Testing the 3 arms on patient level make this study merely an observational/cohort study and prone to the problems known in such study (see below).

Response: Thank you for your review and comment. The study seeks to identify which of the three major HIV treatment delivery models operational in Nigeria will be suitable to deliver PrEP as an intervention to HIV-1 serodiscordant couples. This informed the choice of study design and selection of sites that have recorded high numbers of serodiscordant couples. In addition, eligible and consenting participants were recruited into each model based on the location they access HIV service.

- The current study is in fact a cohort study with 3 different arms/cohorts. As generally known such study is prone to confounding factor problems when attempting to compare between the arms/cohorts. Had the impact of external variables such as age, viral load of partner, region, coital act, use of condom on uptake of PrEP and TasP and on HIV sero-conversion been assessed and incorporate as adjustment factors into the model? This kind of adjustment is needed (for example using Cox regression mode adjusting for those factors instead of simple logrank test), particularly since the analysis suggested the imbalance of these factors across the arms (there were significant differences in the mean age, and the sex, educational level and wealth distribution of the study participants per model). Other factors that need to be considered for the adjustment are the number of unprotected sex acts in the prior month and the number of participants who had sex outside the relationship in the prior month.

Response: Your comment is appreciated. We recognize some of these limitations and have included Cox regression analysis to examine multivariate factors across the models.

- The sample size of the study was calculated based on 2-sided alpha = 5%, when in fact the number of comparisons between arms was 3. It simply suggests that number of comparison was not taking into account at the design level, i.e. no adjustment to the level of alpha. How do you justify this?

Response: Thank you. The sample size formula for comparing two or more proportions was used in the study to determine the minimum sample size per group at 110. After adjusting for attrition the sample size came to 130 participants per group.

- For the survival analysis, the event of interest was the discontinuation of couple’s visit. Did you have any case when only one person of the couple visited? How do you handle such case in term of event or censoring?

Response: Thank you. Yes we had few cases when only one of the couples visited during the follow up visit period. Per protocol such couples were censored to have discontinued the study. They were adjudged as being ineligible to continue the study.

- What was the visit window in all the 3 arms? How frequent was the couples seen/assessed in these 3 different arms? This visit information is sensitive when comparing the KM curves.

Response: The visit window were same for the three models. The couples were seen at month 0, 3, 6, 9, and 12.

- Lines 291-292: ‘The HIV-1 positive incidence before initiation of the study was compared with the HIV-1 incidence at the end of the study per model. The pre-study initiation data at the study site was collected 12 months prior to study initiation’. Apparently the pre-study incidence was collected retrospectively. How did you handle potential bias due to this retrospective collection? How did you ensure the validity of the comparison between pre and end study incidence?

Response: Thank you. The facility HIV records were the source of the retrospective data on seroconversion rate among serodiscordant couples for the pre-study period. The comparison was made pre-study and post-study as incidence per 100 person years.

- Adherence (lines 348-359). ‘At months 3, 6, 9 and 12 follow-up visits, 152 (74.5%), 117 (64.3%), 77 (47.5%) and 71 (32.1%) subjects continued to receive PrEP, respectively …’, as this was a self-reporting adherence, had there any mechanism in the study established to verify this?

Response: Thank you so much for this comment. A number of mechanisms were established in the study to verify self-reported adherence among the participants. Firstly, among the HIV-1 infected partners that are on ART viral load test were conducted to ascertain if they are achieving viral suppression; as expected if they are adherent to their ART. In addition, HIV-1 infected partners ARV drug dispensing records were also monitored for missed appointments, default and lost to follow up. On the other hand, for HIV-1 uninfected partner self reported adherence was verified through use of MEMSCap, a drug dispensing bottle embedded with a microchip to detect the number of times and days participant open the bottle to take PrEP medication. Also, biomarker test for presence of ARvmetabolites was conducted for participants on PrEP.

- Discussion, lines 374-375 stated: ‘Results of the study indicate that although the rate of retention of study participants on PrEP did not differ by model adherence to PrEP differed according to model: …’ yet at the conclusion stated: ’However, client retention was significantly higher in an outpatient model than in the ART clinic and decentralized models.’ How do you explain this discrepancy?

Response: Thank you so much, the correction has been effected.

Reviewer #2: This manuscript describes a demonstration project of PrEP among HIV serodiscordant couples in Nigeria.
Broadly, the work seems like it was reasonably done. The premise of the project is strong. The analyses are hard to follow, however, and important concerns arise.

Major comments:
Comparisons across models. The design of the study is not particularly clear. It seems to be a cohort, with three different models at three locations. In at least one place, these models are described as randomized (Line 136). However, a randomized comparison of n=3 is not a very powerful design and does not permit a robust comparison. There are many factors – staffing of clinic, catchment of clinic, etc. – that undoubtedly influence outcomes in a study like this. The presentation of results and interpretation should recognize this much more. To have done a true robust comparison would have been randomly assigning probably a dozen or more clinics to the different delivery approaches. Instead, simply acknowledging that three models were used and comparisons across them are subject to bias and confounding that is hard to assess is probably fine for a pilot demonstration like this.

Response: Thank you for your review and comment. The study seeks to identify which of the three major HIV treatment delivery models operational in Nigeria will be suitable to deliver PrEP as an intervention to HIV-1 serodiscordant couples. This informed the choice of study design and selection of sites that have recorded high numbers of serodiscordant couples. The comparison is made across the three models, such that each model served as the control for the other two.

Abstract. Per the point above, the results should focus more on the findings and less on the comparisons across groups. The retention and adherence numbers aren’t even described – just p-values – which is exactly the opposite of what is needed.

Response: Thank you, the correction has been effected.

Retention. The retention numbers are difficult to follow, in part because different numbers are presented in different ways (Table 2, Figure 1, Figure 2). Figure 2 in particular looks like terrible retention.

Response: Thank you. We admit that retention in the study declined with time and it is difficult to have same numbers for different time period. The result has been represented in a simpler way in the revision.

HIV incidence. Incident HIV is described as the primary outcome (like 256), which seems impossible – the study could not have been powered, either in each model or in all three combined, to assess HIV incidence. The incidence analyses are difficult to interpret – the text is not clear what is HIV incidence before the study period (and who were these individuals before the study period? Are they beyond the 297 followed?) and what is in the study period. Figure 4 is not helpful, and the analyses associated are terribly underpowered. The authors might just be better off saying there was 1 HIV infection in the 297 couples, in a couple who had done 6 months of ART and stopped PrEP (and whether viral load information was known for the partner living first with HIV).

Response: Thank you for your review comment. The result have been represented for clarity.

Minor comments:
Lines 61-62. The statement that PrEP has reduced HIV at the population level is true, but the placement of this sentence seems off – the authors may be instead referring to clinical trial populations rather than truly at the population level. A reference would be good too.

Response: Thank you for pointing this out, references has been cited.

Line 111. Why were pregnant and lactating women excluded?

Response: Thank you. Pregnant and lactating women were excluded because the teratogenic effect and effect on the breastfeeding child of the PrEP medication tenofovir was not well known at the time of the study.

Line 176. What is the Partners PrEP Study approach?

Response: Thank you. The Partners PrEP Study is a double-blind, placebo-controlled, phase II clinical trial to assess the safety and efficacy of oral PrEP for the prevention of HIV infection, using antiretroviral medication tenofovir (TDF), either alone or in combination with emtricitabine (FTC/TDF). The trial demonstrated the benefit of tenofovir and recommended the discontinuation of placebo. The Partners PrEP Study approach is a group of demonstration study on how to deliver PrEP effectively for populations at greatest risk for HIV. The study is funded by Bill & Melinda Gates Foundation. The drugs are donated by Gilead Sciences Incorporated.
