# Supplementary material for: Effectiveness of three delivery models for promoting access to pre-exposure prophylaxis in HIV-1 serodiscordant couples in Nigeria
Source: PLoS One. 2022 May 5;17(5):e0268011. doi: 10.1371/journal.pone.0268011 (PMC9070899; doi:10.1371/journal.pone.0268011)
Supplement: Attachment — Submitted filename: Response to Reviewers.docx [file pone.0268011.s007.docx]

Rebuttal Letter

Response to Reviewers

Reviewer #1: Some of the major not properly addressed, namely:

- The experimental unit of this study was site as randomization was done at site level. However there was only a single site for each treatment arm. This essentially makes the study arm un-testable utilizing the randomization (i.e. no possible inference in a randomized setting based on sampling unit). Testing the 3 arms on patient level make this study merely an observational/cohort study and prone to the problems known in such study (see below).
Response: Thank you for your review and comment. The study seeks to identify which of the three major HIV treatment delivery models operational in Nigeria will be suitable to deliver PrEP as an intervention to HIV-1 serodiscordant couples. This informed the choice of study design and selection of sites that have recorded high numbers of serodiscordant couples. In addition, eligible and consenting participants were recruited into each model based on the location they access HIV service.

Re: The above response does not address the comments appropriately. In the current study, your randomization can not be used as a basis of inference. Please address this question.

Response: Thank you for your review comment, the study groups have been amended as cohorts.

- Original comment: The current study is in fact a cohort study with 3 different arms/cohorts. As generally known such study is prone to confounding factor problems when attempting to compare between the arms/cohorts. Had the impact of external variables such as age, viral load of partner, region, coital act, use of condom on uptake of PrEP and TasP and on HIV sero-conversion been assessed and incorporate as adjustment factors into the model? This kind of adjustment is needed (for example using Cox regression mode adjusting for those factors instead of simple logrank test), particularly since the analysis suggested the imbalance of these factors across the arms (there were significant differences in the mean age, and the sex, educational level and wealth distribution of the study participants per model). Other factors that need to be considered for the adjustment are the number of unprotected sex acts in the prior month and the number of participants who had sex outside the relationship in the prior month.

Response: Your comment is appreciated. We recognize some of these limitations and have included Cox regression analysis to examine multivariate factors across the models.

Re: This comment is not addressed properly. The Cox model which is presented in the current article does not do any comparison between three major HIV treatment delivery models.

Response: Thank you. A Cox survival analysis with adjusted Hazard ratio has been conducted to compare among the three treatment groups, please refer table 4.

- Original comment: The sample size of the study was calculated based on 2-sided alpha = 5%, when in fact the number of comparisons between arms was 3. It simply suggests that number of comparison was not taking into account at the design level, i.e. no adjustment to the level of alpha. How do you justify this?

Response: Thank you. The sample size formula for comparing two or more proportions was used in the study to determine the minimum sample size per group at 110. After adjusting for attrition the sample size came to 130 participants per group.

Re: This comment is not addressed properly. Justification of not penalizing alpha despite multiple comparisons is needed.

Response: Thank you. A Bonferroni correction factor of 3 has been applied to the alpha to account for the three treatment groups.

- Original comment(Lines 291-292): ‘The HIV-1 positive incidence before initiation of the study was compared with the HIV-1 incidence at the end of the study per model. The pre-study initiation data at the study site was collected 12 months prior to study initiation’.
Apparently the pre-study incidence was collected retrospectively. How did you handle potential bias due to this retrospective collection? How did you ensure the validity of the comparison between pre and end study incidence?

Response: Thank you. The facility HIV records were the source of the retrospective data on seroconversion rate among serodiscordant couples for the pre-study period. The comparison was made pre-study and post-study as incidence per 100 person years.

Re: This comment is not addressed properly. Data collected retrospectively and data collected prospectively are 2 very different data in term of quality. This should be made clear in the article including its consequence.

Response: Thank you. Data collected retrospectively before the study are prone to data quality issues such as completeness, accuracy and precision of operational definitions.
